# Tranexamic acid for neurological outcomes and mortality in adults with hemorrhagic acute brain injury: an updated systematic review and meta-analysis of randomized controlled trials

**DOI:** 10.3389/fneur.2026.1902987

**Published:** 2026-07-31

**Authors:** Zhengyi Zhang, Zilin He, Kunlan Long

**Affiliations:** 1 School of Clinical Medicine, Chengdu University of Traditional Chinese Medicine, Chengdu, China; 2 Department of Critical Care Medicine, Hospital of Chengdu University of Traditional Chinese Medicine, Chengdu, China

**Keywords:** acute brain injury, meta-analysis, mortality, neurological prognosis, tranexamic acid

## Abstract

**Background:**

Hemorrhagic acute brain injury—encompassing traumatic brain injury, spontaneous intracerebral hemorrhage, and subarachnoid hemorrhage—carries substantial morbidity and mortality. Active hemorrhage and hematoma progression constitute shared pathological mechanisms across these conditions. As a lysine analog anti-fibrinolytic, tranexamic acid (TXA) has demonstrated mortality reduction in extracranial bleeding; however, its efficacy and safety in intracranial hemorrhagic lesions remain inconclusive. Prior systematic reviews predominantly targeted single disease categories, inadequately assessed core outcomes such as all-cause mortality and neurological functional recovery, and seldom applied the Risk of Bias 2 tool or GRADE framework for methodological appraisal. Therefore, an updated meta-analysis is warranted to re-evaluate the overall clinical value of TXA in hemorrhagic acute brain injury.

**Objective:**

To evaluate the effect of TXA on neurological outcomes and all-cause mortality in adults with acute brain injury.

**Methods:**

This meta-analysis was registered with PROSPERO (CRD420261400869). In accordance with the PRISMA 2020 statement, we systematically searched PubMed, EMBASE, Scopus, Web of Science, and the Cochrane Library from inception to April 2026. Eligible studies were randomized controlled trials comparing TXA with placebo in hospitalized adults with acute brain injury, including traumatic brain injury, spontaneous intracranial hemorrhage, and subarachnoid hemorrhage. Risk of bias was assessed using the RoB 2 tool, the certainty of evidence was evaluated using the GRADE framework, pooled effect estimates were generated using a random-effects model, heterogeneity was quantified using the *I*^2^ statistic, and robustness was tested through leave-one-out sensitivity analysis. The primary outcomes were neurological outcomes and all-cause mortality; secondary outcomes were thromboembolic events and intracranial rebleeding.

**Results:**

Seven high-quality randomized controlled trials comprising over 12,000 patients were included. Random-effects model analyses showed no statistically significant effect of TXA on short-term mortality (RR 0.93, 95% CI 0.86–1.00) or long-term mortality (RR 1.02, 95% CI 0.91–1.13). For neurological outcomes, TXA neither improved the proportion of patients with functional independence (RR 0.97, 95% CI 0.92–1.03) nor reduced the risk of functional dependence or death (RR 1.01, 95% CI 0.96–1.07). For rebleeding, the pooled estimate showed no statistically significant effect (RR 0.81, 95% CI 0.59–1.11), with substantial between-study heterogeneity (*I*^2^ = 72%). The current evidence is inconclusive regarding a protective effect of TXA on rebleeding. The incidence of thromboembolic events did not differ significantly between groups (RR 1.17, 95% CI 0.90–1.52). Leave-one-out sensitivity analyses indicated that the primary outcomes were robust.

**Conclusion:**

TXA did not improve all-cause mortality or neurological outcomes in hemorrhagic acute brain injury, and the evidence for its effect on rebleeding remains inconclusive.

**Systematic review registration:**

PROSPERO, CRD420261400869.

## 1 Background

Hemorrhagic acute brain injury—encompassing traumatic brain injury, spontaneous intracerebral hemorrhage, and aneurysmal subarachnoid hemorrhage—represents a leading cause of death and long-term neurological disability worldwide (1, 2). Although these conditions differ in etiology, active hemorrhage and hematoma expansion constitute a shared core pathological mechanism that drives secondary brain injury through mass effect, elevated intracranial pressure, and neurotoxic cascades, directly determining clinical outcomes (3, 4).

Tranexamic acid (TXA) is a synthetic lysine analog that competitively blocks the binding of plasminogen to fibrin, thereby inhibiting fibrinolysis and stabilizing formed blood clots (5). The CRASH-2 trial demonstrated that TXA reduces mortality in patients with traumatic bleeding, and individual patient data analyses revealed a marked time-dependent treatment effect, with earlier administration conferring greater benefit (6, 7). This evidence has substantially advanced the use of TXA in acute hemorrhagic conditions. European guidelines recommend prompt TXA administration in patients with ongoing bleeding or at high risk of severe hemorrhage (8). However, the efficacy and safety of TXA in intracranial hemorrhagic lesions remain inconclusive, and the European Stroke Organization and neurosurgical societies maintain a cautious stance regarding its application in intracranial bleeding (9).

Recent randomized controlled trials have reported inconsistent efficacy findings for TXA across subtypes of hemorrhagic acute brain injury. The CRASH-3 trial showed that TXA did not significantly reduce overall head injury–related mortality, yet suggested potential benefit in patients with mild-to-moderate traumatic brain injury (10). The TICH-2 trial indicated that TXA modestly reduced the risk of early hematoma expansion but did not improve 90-day functional outcomes (11). The STOP-AUST and STOP-MSU trials also found no clear clinical benefit regarding hematoma control or neurological recovery (12, 13). The ULTRA trial confirmed that ultra-early short-course TXA reduced rebleeding in aneurysmal subarachnoid hemorrhage, yet did not improve long-term functional prognosis (14). Collectively, these findings suggest that although TXA may attenuate acute bleeding through anti-fibrinolysis, clinical outcomes in hemorrhagic acute brain injury are not determined by bleeding volume alone, and its hemostatic effect may not translate directly into improved patient-important outcomes.

Existing systematic reviews and meta-analyses have largely been confined to single disease subtypes, and an integrated analysis spanning hemorrhagic acute brain injury remains unavailable, precluding an evaluation of the overall clinical value of TXA from the standpoint of shared pathological mechanisms (15–18). Furthermore, outcome definitions varied across studies, with some employing surrogate endpoints such as hematoma expansion or rebleeding while all-cause mortality and neurological outcomes received insufficient assessment. As high-quality RCTs have been published in recent years, the currency of evidence in prior reviews has become limited. Meanwhile, many studies did not adopt the RoB 2 and GRADE frameworks for systematic methodological appraisal (19, 20). Accordingly, by stringently including high-quality RCTs and applying the RoB 2, this study integrates the effects of TXA on mortality, functional outcomes, and safety endpoints in hemorrhagic acute brain injury to provide high-certainty evidence for hemostatic strategies in neuro-critical care, clarify the sources of heterogeneity in TXA efficacy and the characteristics of populations most likely to benefit, and guide the optimization of future RCT designs and the formulation of precision hemostatic protocols.

## 2 Methods

### 2.1 Materials

This systematic review and meta-analysis was conducted and reported in strict accordance with the methodological standards of the Cochrane Handbook for Systematic Reviews of Interventions (21) and the Preferred Reporting Items for Systematic Reviews and Meta-Analyses (PRISMA) 2020 statement (22). The study protocol was registered with the International Prospective Register of Systematic Reviews (PROSPERO; registration number: CRD420261400869). The complete PRISMA 2020 checklist is provided in Supplementary file 1.

### 2.2 Literature search

Two investigators independently performed the literature search and screening across five databases: The Cochrane Library (Cochrane Central Register of Controlled Trials and Cochrane Database of Systematic Reviews), EMBASE, PubMed, Web of Science, and Scopus. The search period extended from inception to April 2026, with language restricted to English. A combination of Medical Subject Headings (MeSH) terms and free-text words was employed. Search terms included “tranexamic acid,” “TXA,” “antifibrinolytic,” “brain injury,” “traumatic brain injury,” “intracerebral hemorrhage,” “subarachnoid hemorrhage,” and “randomized controlled trial” (the complete search strategy is detailed in Supplementary file 2). Two investigators independently screened titles and abstracts for initial eligibility, followed by full-text review of potentially eligible records. Disagreements were resolved through discussion and consensus, with arbitration by a third investigator when necessary. We included high-quality randomized controlled trials comparing TXA with placebo in adults with acute brain injury that reported neurological outcomes and all-cause mortality. We excluded studies of low methodological quality, conference abstracts, gray literature, non-randomized controlled trials, secondary data analyses, studies not involving acute brain injury, those with missing key outcome data, and animal experiments.

### 2.3 Inclusion and exclusion criteria

This meta-analysis included studies meeting all of the following criteria. Inclusion criteria: (1) randomized controlled trial design of high methodological quality; (2) adult patients (aged ≥18 years) with intracranial hemorrhage from any cause, including spontaneous intracranial hemorrhage, subarachnoid hemorrhage, traumatic intracranial hemorrhage, and other acute brain injuries characterized by active intracranial bleeding (23); (3) intervention with TXA (dose, route of administration, and treatment duration unrestricted); (4) comparator receiving placebo, normal saline, or standard care without TXA or other systemic anti-fibrinolytic agents; (5) reporting of neurological outcomes and/or all-cause mortality, with extractable data from the original article, appendices, or Supplementary material.

Exclusion criteria: (1) non-randomized controlled trial design, or studies with prohibitively low methodological quality precluding accurate estimation of intervention effects; (2) study populations without acute brain injury (e.g., isolated extracranial trauma, ischemic stroke without hemorrhagic transformation, chronic subdural hematoma, unruptured aneurysm); (3) studies in which the intervention group received TXA combined with other systemic anti-fibrinolytics and the independent effect of TXA could not be isolated; (4) studies with missing key outcome data unavailable from the full text, appendices, or Supplementary material; (5) pediatric populations (age <18 years) or studies in which informed consent was refused by patients or their families; (6) non-randomized study designs, including observational studies, secondary analyses, cohort studies, case series, reviews, meta-analyses, conference abstracts, protocols, and animal experiments; (7) duplicate publications, with only the most complete or largest dataset retained.

### 2.4 Data extraction

To ensure standardized data extraction, we designed and used a predefined structured data extraction form, which was completed independently by two investigators (ZZ and HZ). The following items were extracted: first author, year of publication, country, and article title; baseline characteristics of the study population (sample size, sex distribution, and etiology of intracranial hemorrhage); intervention details for the TXA group (dose, route of administration, timing of administration, and treatment duration); and comparator details for the control group (specifics of placebo, normal saline, or standard care regimens). Additionally, treatment outcomes and data for primary and secondary outcomes were systematically extracted for both groups. For missing key data, we contacted the corresponding authors via email to request supplementary information, with multiple rounds of follow-up conducted when necessary to ensure data completeness.

### 2.5 Outcomes

The pre-specified primary outcomes were as follows: (1) All-cause mortality, stratified by follow-up duration. Short-term mortality was defined as mortality at ≤30 days (or the nearest available time point, such as 28 days); long-term mortality was defined as mortality at the longest follow-up time point beyond 30 days (e.g., 3, 6, or 12 months). When a single study reported multiple time points, data for the two standard time points described above were preferentially extracted for analysis. (2) Neurological outcomes (assessed using the modified Rankin Scale [mRS], with the proportions of functional independence and functional dependence or death reported). Functional independence was defined as an mRS score of 0–3, corresponding to no disability through moderate disability with preserved capacity for basic independent living (encompassing no symptoms; no significant disability despite symptoms, with preserved ability to perform daily activities; slight disability with the ability to handle personal affairs without assistance; and moderate disability with the ability to walk without assistance). Functional dependence was defined as an mRS score of 4-5, indicating moderate-to-severe disability precluding independent living (11).

Secondary outcomes included the incidence of thromboembolic events (encompassing ischemic stroke, deep vein thrombosis, pulmonary embolism, myocardial infarction, and other thromboembolic events) and the rate of rebleeding (including intracranial rebleeding, hematoma expansion, and extracranial bleeding events requiring intervention). An outcome was included in the meta-analysis for pooled estimation only if it was reported in ≥3 independent studies.

### 2.6 Risk of bias

Two investigators (ZZ and HZ) independently assessed risk of bias in the included randomized controlled trials using the Cochrane Risk of Bias 2 (RoB 2) tool (24), with the assessment focused on the two primary outcomes: all-cause mortality and neurological outcomes. The RoB 2 tool comprises five key domains: (1) bias arising from the randomization process; (2) bias due to deviations from intended interventions; (3) bias due to missing outcome data; (4) bias in measurement of the outcome; and (5) bias in selection of the reported result. Based on the domain-level assessments, each study received an overall risk-of-bias judgment of low risk, some concerns, or high risk of bias. Disagreements between the two assessors were resolved through discussion and consensus; if agreement could not be reached, a third independent investigator (LK) arbitrated to achieve final consensus.

### 2.7 Assessment of certainty of evidence

Two investigators (ZZ and HZ) independently assessed the certainty of evidence for each primary and secondary outcome using the GRADE framework (20, 25). The GRADEpro GDT software^1^ was used to construct Summary of Findings tables. For randomized controlled trials, the certainty of evidence started at high and was downgraded by one or two levels for serious or very serious concerns across the following five domains: (1) risk of bias, based on the RoB 2 assessments; (2) inconsistency, indicated by substantial statistical heterogeneity (*I*^2^ > 50%) or divergent effect estimates across studies; (3) indirectness, relating to differences between the PICO elements of the included studies and the review question; (4) imprecision, judged by the width of the 95% confidence interval and whether the optimal information size was reached; and (5) publication bias, assessed descriptively through funnel plots given the limited number of included studies (<10). The final certainty was rated as high, moderate, low, or very low. Disagreements were resolved through discussion, with arbitration by a third investigator (LK) when necessary.

### 2.8 Statistical analysis

All statistical analyses were performed using R software (version 4.4.2). For dichotomous outcomes, relative risks (RRs) and 95% confidence intervals (CIs) were calculated. Pooled effect estimates were generated using fixed-effects models with the Mantel–Haenszel method and random-effects models with the DerSimonian–Laird method. Between-study heterogeneity was assessed using Cochran’s *Q* test and the *I*^2^ statistic. Significant statistical heterogeneity was considered present when *I*^2^ > 50% and Cochran’s *Q* test yielded *p* < 0.10.

Given the anticipated clinical heterogeneity across included studies in population characteristics (etiology, injury severity, geographic region), intervention parameters (dose, time window of administration, treatment duration), and outcome definitions (follow-up time points, assessment instruments), we uniformly employed random-effects models (DerSimonian–Laird method) as the primary analysis for all outcomes, with fixed-effects models (Mantel–Haenszel method) used as sensitivity analyses to verify the robustness of the findings. The DerSimonian–Laird method is a conventional and widely used approach for random-effects meta-analysis. When the number of included studies is relatively small (fewer than 10), this estimator may produce relatively wide confidence intervals. Therefore, we have exercised particular caution in interpreting the pooled estimates, especially for outcomes with substantial heterogeneity. Additionally, to assess the stability of the results, leave-one-out (LOO) sensitivity analyses were conducted using OpenMetaAnalyst software; each individual study was sequentially omitted and the meta-analysis was repeated to observe changes in the pooled effect estimates and confidence intervals, thereby evaluating the influence of individual studies on the overall results. As fewer than 10 studies were included, funnel plots were assessed descriptively only. All statistical tests were two-sided, and a *p* < 0.05 was considered statistically significant.

## 3 Results

### 3.1 Literature search

**Figure 1:**
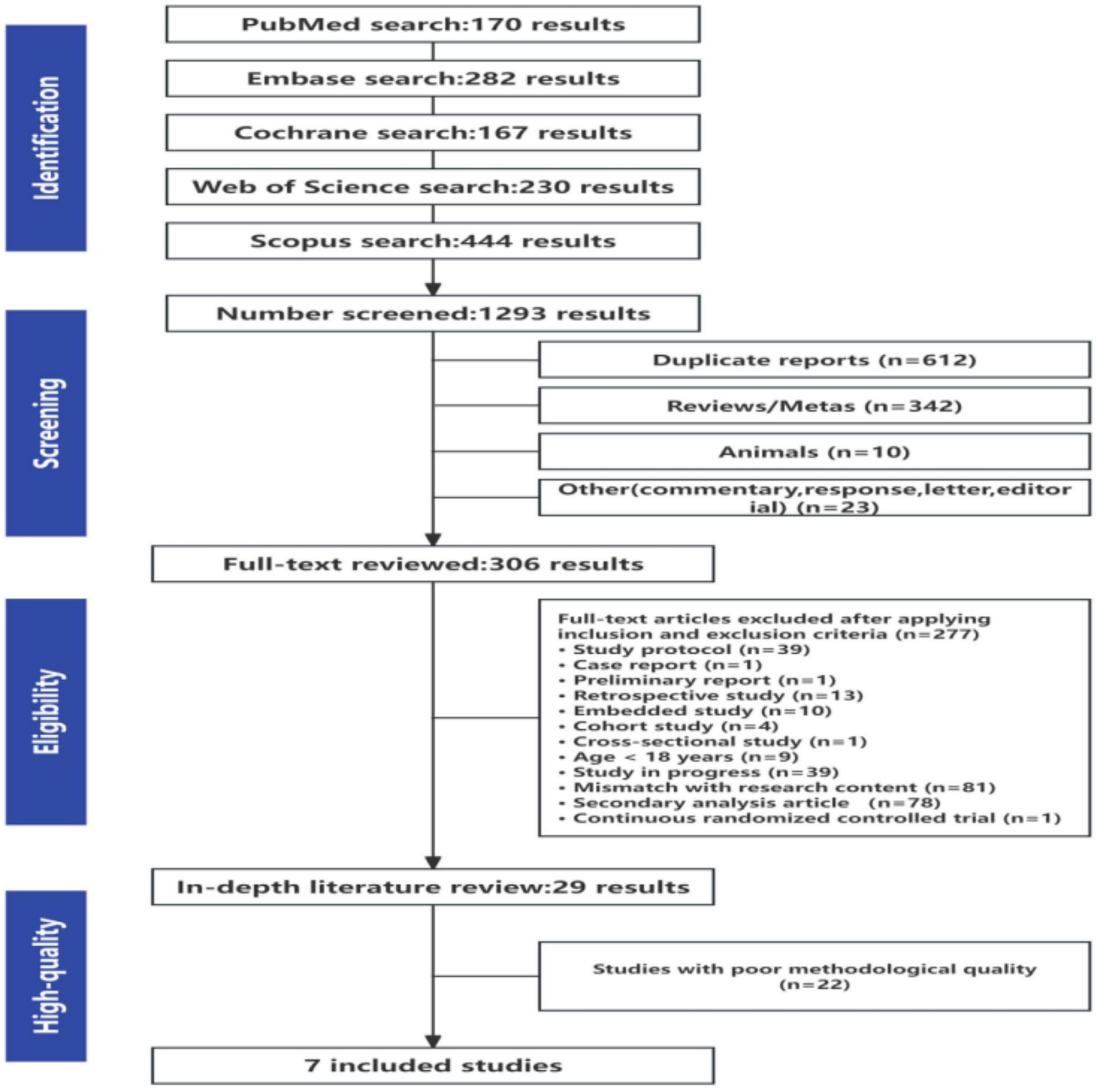
PRISMA flow diagram for studies included in and excluded from the meta-analysis.

Independent searches of five databases yielded 1,293 records. After deduplication (*n* = 612), 681 records underwent title and abstract screening, of which 375 were excluded. Full-text review was performed for 306 potentially eligible records; 277 studies were excluded for not meeting inclusion criteria (reasons included non-randomized controlled trial design, ongoing studies, protocols, and other types). The remaining 29 articles underwent in-depth full-text assessment and methodological quality evaluation. Ultimately, 7 randomized controlled trials with low risk of bias or some concerns were included in this analysis (10–14, 26, 27). The study selection process is detailed in Figure 1.

### 3.2 Study characteristics

Seven randomized controlled trials, published between 1984 and 2024, were included (10–14, 26, 27). These studies encompassed three hemorrhagic acute brain injury subtypes: traumatic brain injury, intracerebral hemorrhage, and aneurysmal subarachnoid hemorrhage, with a combined total of over 12,000 patients. Study designs evolved from early single-center open-label trials to modern international multicenter double-blind placebo-controlled trials. The predominant intervention regimen was a 1 g intravenous bolus followed by a 1 g maintenance infusion over 8 h; only the Vermeulen study (26) employed individualized titration for up to 4 weeks. The time window for drug administration ranged from 2 to 72 h across studies. Primary endpoints included the modified Rankin Scale, Glasgow Outcome Scale, and surrogate endpoints such as hematoma expansion. Two large phase III trials (10, 11) contributed approximately 90% of the total sample size. Detailed characteristics of the included studies are presented in Table 1.

**Table 1:** Characteristics of included studies on tranexamic acid for acute brain injury.

Study	Trial	Country	Design	Population	*n* (TXA/C)	Time window	TXA regimen	Primary outcome
Vermeulen et al. 1984 (26)	—	Rotterdam, Amsterdam, Glasgow, London	RPC	Aneurysmal SAH	241/238	≤72 h	6 g/d → 4 g/d IV; max 4 wk.^†^	GOS (3 mo)
Sprigg et al. 2018 (11)	TICH-2	12 countries	Phase 3 DB RPC	Spontaneous ICH	1,161/1,164	≤8 h	1 g IV bolus + 1 g IV 8 h	mRS shift (90 d)
CRASH-3 Trial Collaborators et al. 2019 (10)	CRASH-3	29 countries	Phase 3 DB RPC	TBI (GCS ≤ 12 or bleed on CT)	4,613/4,514	≤3 h	1 g IV bolus + 1 g IV 8 h	Head injury death (28 d)
Meretoja et al. 2020 (12)	STOP-AUST	Australia, Finland, Taiwan	Phase 2 DB RPC	Spot-sign positive ICH	50/50	≤4.5 h	1 g IV bolus + 1 g IV 8 h	HE >33% or >6 mL (24 h)
Post et al. 2020 (14)	ULTRA	Netherlands	Phase 2 OL RC	Aneurysmal SAH	480/475	≤24 h	1 g IV + 1 g q8h	mRS shift (6 mo)
Polymeris et al. 2023 (27)	TICH-NOAC	Switzerland	Phase 2 DB RPC	NOAC-related ICH	32/31	≤12 h	1 g IV bolus + 1 g IV 8 h	HE ≥33% or ≥6 mL (24 h)
Yassi et al. 2024 (13)	STOP-MSU	5 countries	Phase 2 DB RPC	Spontaneous ICH	101/97	≤2 h	1 g IV bolus + 1 g IV 8 h	HE ≥33% or ≥6 mL (24 h)

C, control group (patients receiving placebo, normal saline, or standard care without TXA); DB, double-blind; GOS, Glasgow Outcome Scale; HE, hematoma expansion; ICH, intracerebral hemorrhage; mo, months; NOAC, non–vitamin K antagonist oral anticoagulant; OL, open-label; RC, randomized controlled; RPC, randomized placebo-controlled; SAH, subarachnoid hemorrhage; TBI, traumatic brain injury; TXA, tranexamic acid. ^†^Dose tapered from 6 g/d (1 g q4h) in week 1 to 4 g/d (1 g q6h) thereafter. In two centers, IV treatment was switched to oral 6 g/d (1.5 g q6h) after week 2. Treatment was discontinued at aneurysm surgery, alternative diagnosis, or thromboembolic events.

### 3.3 Risk of bias

**Figure 2:**
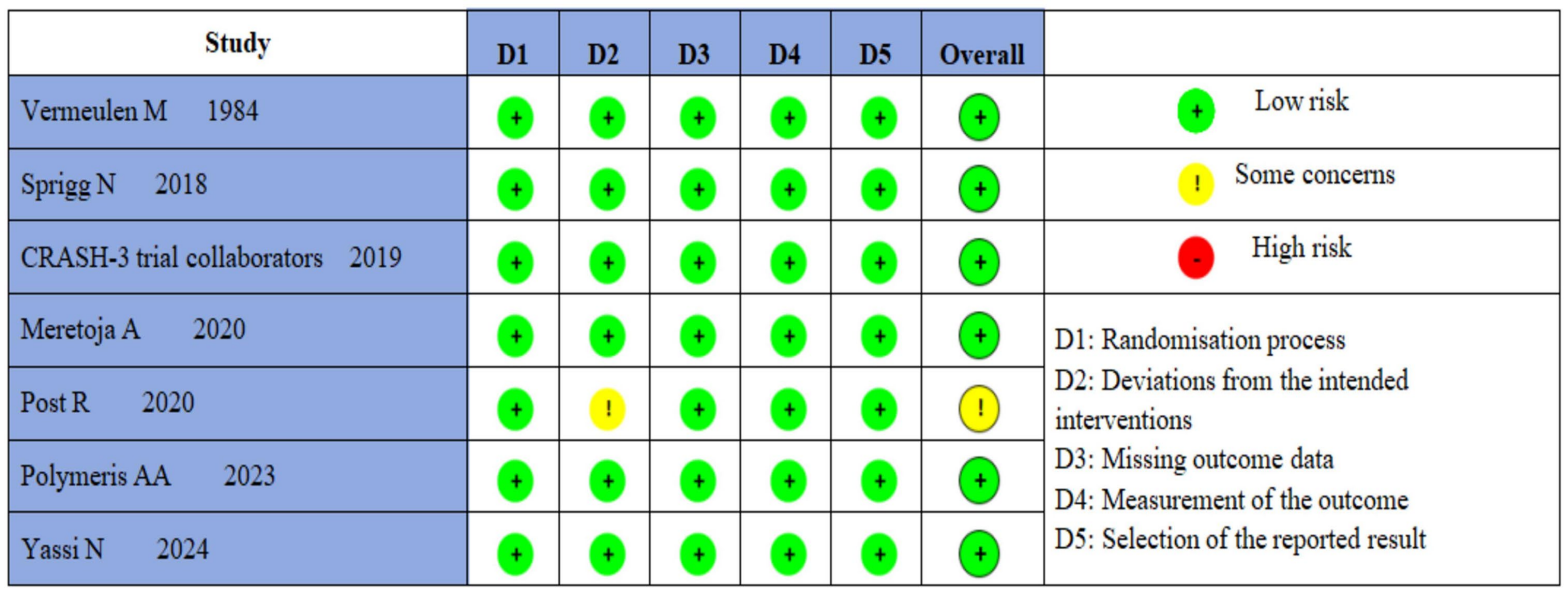
Risk of bias 2 summary figure.

Risk of bias in the 7 included randomized controlled trials (10–14, 26, 27) was assessed using the Cochrane Risk of Bias 2 (RoB 2) tool. The overall risk of bias was generally low. All studies were rated as low risk in four domains: randomization process, missing outcome data, outcome measurement, and selective reporting. In the domain of deviations from intended interventions, one study (14) was rated as having some concerns due to insufficient reporting of intervention implementation details and adherence data; the remaining 6 studies were rated as low risk. For overall risk of bias, 6 studies (10–13, 26, 27) were judged to be at low risk, and 1 study was judged to have some concerns. The risk of bias assessment results are detailed in Figure 2 (Supplementary file 3).

### 3.4 Mortality

**Figure 3:**
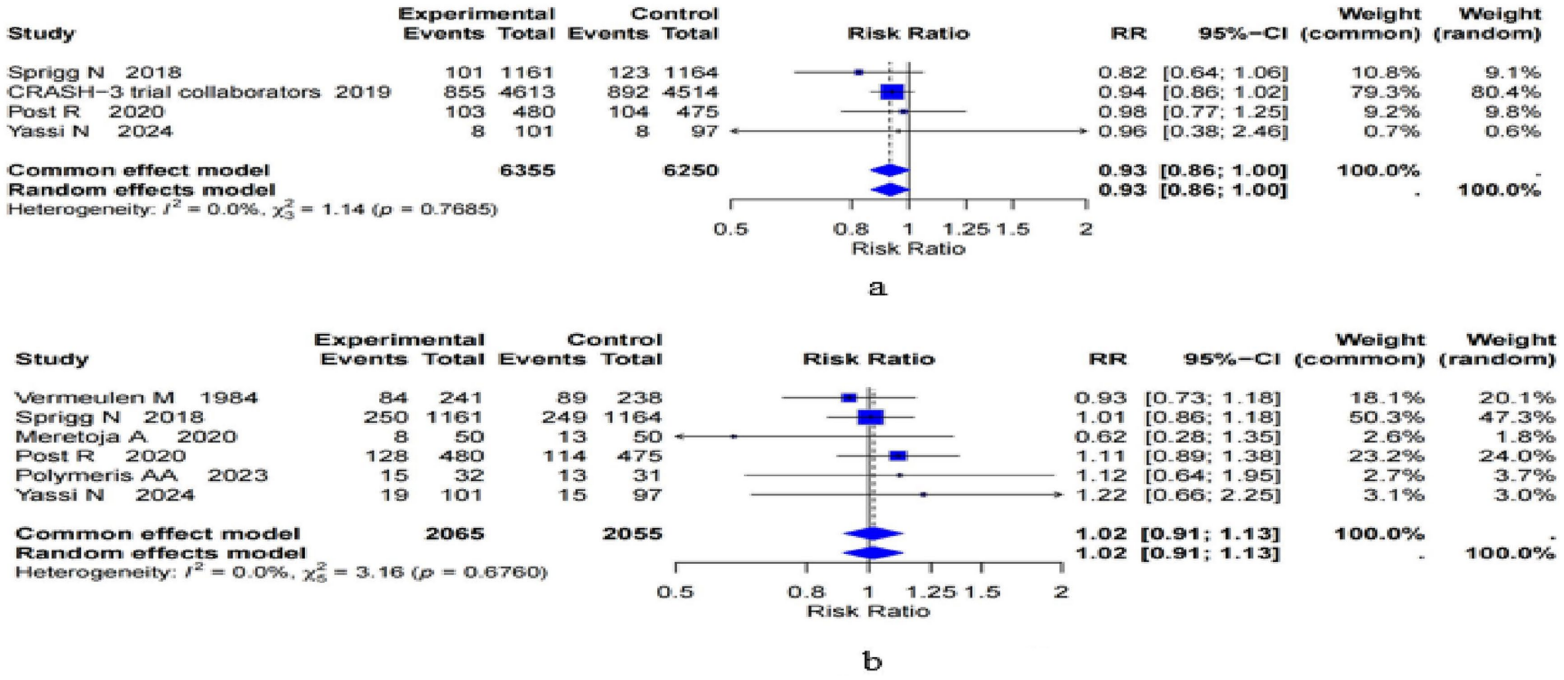
Forest plot of the rate of mortality. **(a)** Short-term mortality. **(b)** Long-term mortality.

For short-term mortality, four randomized controlled trials (10, 11, 13, 14) comprising 6,355 patients in the TXA group and 6,250 in the control group were included. Pooled analysis using a random-effects model showed that TXA was associated with a reduction in short-term mortality, but the difference was not statistically significant (RR 0.93, 95% CI 0.86–1.00; Figure 3a). The upper limit of the confidence interval abutted the line of no effect, suggesting that any protective trend is uncertain at the current sample size and may reflect sampling error. No significant heterogeneity was observed across studies (*I*^2^ = 0%, *χ*^2^ = 1.14, *p* = 0.769). Leave-one-out sensitivity analysis showed that after sequentially excluding each study, the pooled effect estimates ranged from 0.90 to 0.94, with no substantial changes in the 95% CIs and heterogeneity remaining at 0%. The fixed-effects model yielded results consistent with the primary analysis (RR 0.93, 95% CI 0.86–1.00), indicating robust findings. Funnel plots showed no obvious asymmetry; however, given that only 4 studies were included, the interpretation of publication bias assessment should be cautious.

About long-term mortality, six randomized controlled trials (11–14, 26, 27) comprising 2,065 patients in the TXA group and 2,055 in the control group were included. Pooled analysis using a random-effects model showed that TXA had no significant effect on long-term mortality (RR 1.02, 95% CI 0.91–1.13; Figure 3b). Low heterogeneity was observed across studies (*I*^2^ = 0%, *χ*^2^ = 3.16, *p* = 0.676). Leave-one-out sensitivity analysis showed that after sequentially excluding each study, the pooled effect estimates ranged from 0.99 to 1.04, all 95% CIs crossed the line of no effect, and *I*^2^ remained at 0%. The fixed-effects model yielded results consistent with the primary analysis (RR 1.02, 95% CI 0.91–1.13), indicating that the results were stable. Funnel plots showed no obvious asymmetry; however, given the limited number of included studies, the assessment of publication bias should be interpreted with caution.

### 3.5 Neurological outcomes

**Figure 4:**
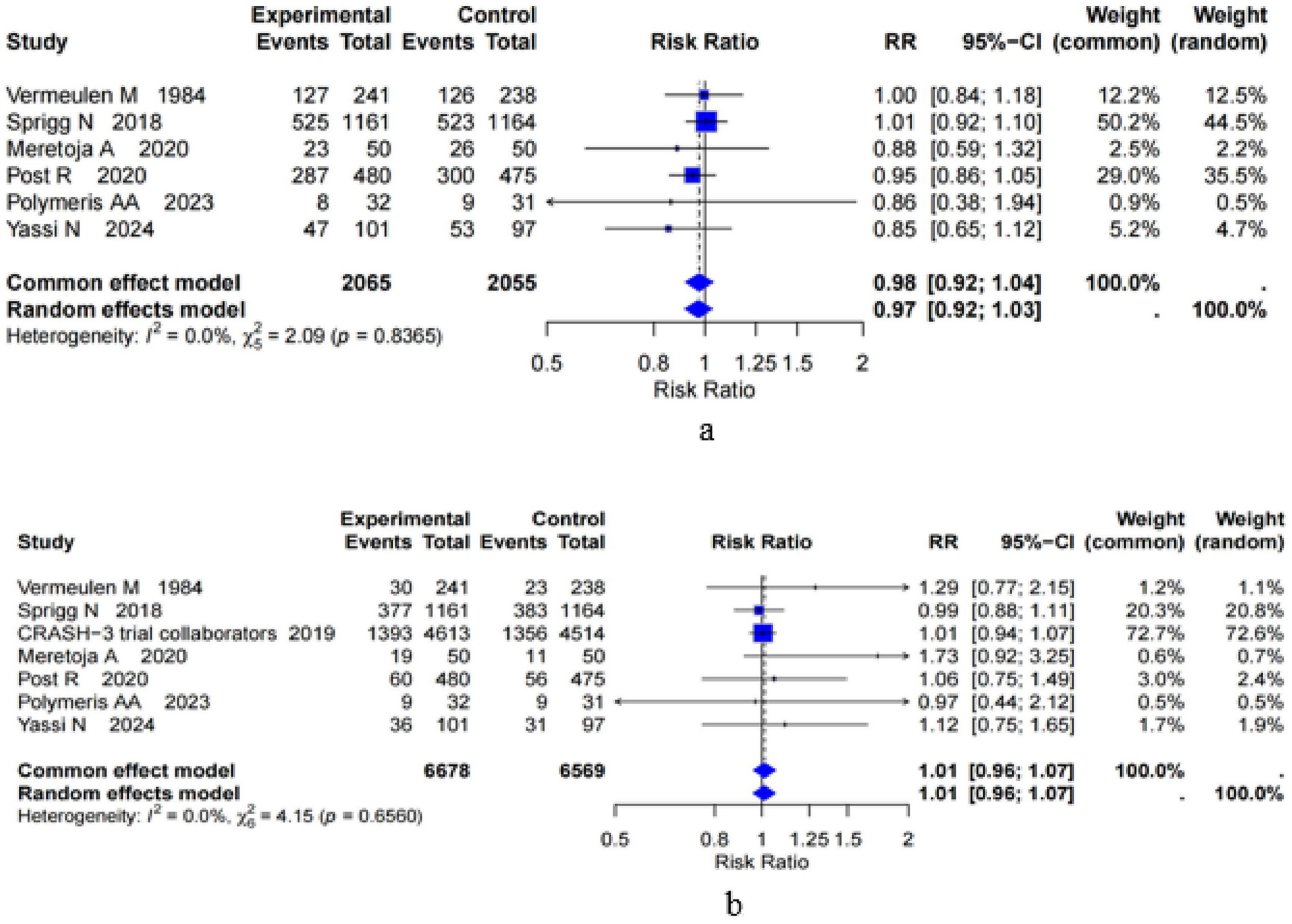
Forest plot of neurological outcomes. **(a)** Functional independence. **(b)** Functional dependence.

According functional independence, six studies (11–14, 26, 27) comprising 2,065 patients in the TXA group and 2,055 in the control group reported functional independence outcomes. Pooled analysis using a random-effects model showed that TXA did not increase the proportion of patients achieving functional independence (RR 0.97, 95% CI 0.92–1.03; Figure 4a). No significant heterogeneity was observed (*I*^2^ = 0%, *χ*^2^ = 2.09, *p* = 0.837), indicating consistent findings across studies. Results from the fixed-effects model were essentially unchanged (RR 0.98, 95% CI 0.92–1.04), further supporting these findings. Leave-one-out sensitivity analysis showed that pooled effect estimates ranged from 0.94 to 0.99, with none indicating a significant improvement in functional independence with TXA, suggesting that the overall conclusion was not dependent on any single study. Funnel plots showed no obvious asymmetry.

According functional dependence, Seven studies (10–14, 26, 27) comprising 6,678 patients in the TXA group and 6,569 in the control group reported functional dependence outcomes. Consistent with the functional independence results, pooled analysis using a random-effects model showed no significant reduction in the risk of functional dependence with TXA (RR 1.01, 95% CI 0.96–1.07; Figure 4b). Very low heterogeneity was observed across studies (*I*^2^ = 0%, *χ*^2^ = 4.15, *p* = 0.656), and fixed-effects model results were consistent with the primary analysis (RR 1.01, 95% CI 0.96–1.07), indicating that this outcome was stable across different statistical methods. Leave-one-out sensitivity analysis showed that pooled effect estimates remained between 1.01 and 1.03 after sequentially excluding individual studies, with no directional change, indicating that TXA neither increased nor decreased the risk of functional dependence. Funnel plots showed no clear asymmetry; however, given the limited number of included studies, potential publication bias should be interpreted with caution.

### 3.6 Rebleeding

**Figure 5:**
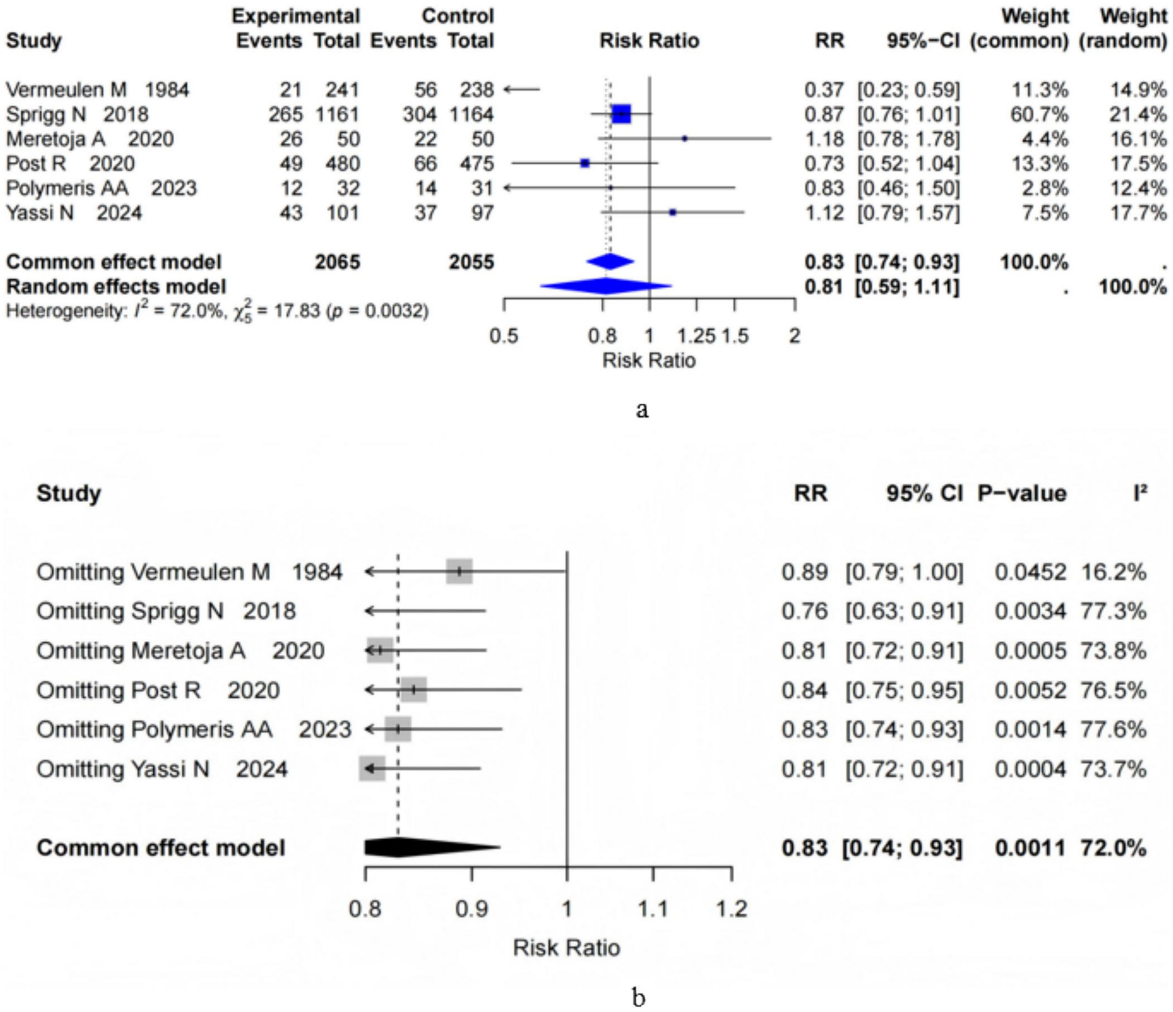
Forest plot of rebleeding. **(a)** Rebleeding. **(b)** Leave-one-out sensitivity analysis for rebleeding.

Six studies (11–14, 26, 27) comprising 2,065 patients in the TXA group and 2,055 in the control group reported this outcome. Pooled analysis using a random-effects model showed that TXA did not significantly reduce the risk of rebleeding (RR 0.81, 95% CI 0.59–1.11; Figure 5a). Substantial heterogeneity was observed across studies (*I*^2^ = 72.0%, *χ*^2^ = 17.83, *p* = 0.003), indicating variation in treatment effects between studies. Under the fixed-effects model, pooled results showed that TXA was associated with a reduced risk of rebleeding (RR 0.83, 95% CI 0.74–0.93). However, this outcome showed substantial model dependency: the fixed-effects model yielded a statistically significant result, whereas the random-effects model produced a confidence interval that crossed the line of no effect. According to the Cochrane Handbook for Systematic Reviews of Interventions, when substantial clinical heterogeneity is present (*I*^2^ = 72%) and cannot be adequately explained through subgroup analysis, the random-effects model provides a more conservative estimate. Therefore, the random-effects model result was adopted as the primary interpretation, leading to the conclusion that current evidence is insufficient to support that TXA reliably reduces the risk of intracranial rebleeding.

Sensitivity analysis showed that after sequentially excluding individual studies, pooled effect estimates ranged from 0.76 to 0.89. With the exception of the analysis excluding the Vermeulen study (26), which yielded a result approaching statistical non-significance (RR 0.89, 95% CI 0.79–1.00), most other scenarios indicated a reduced risk of rebleeding, suggesting that the overall direction of effect was relatively consistent (Figure 5b). However, the statistical significance and magnitude of the effect remained influenced by individual studies and heterogeneity. Funnel plots showed no obvious asymmetry; however, given the limited number of included studies and the substantial between-study heterogeneity, publication bias and small-study effects should be assessed with caution.

### 3.7 Thromboembolic events

**Figure 6:**
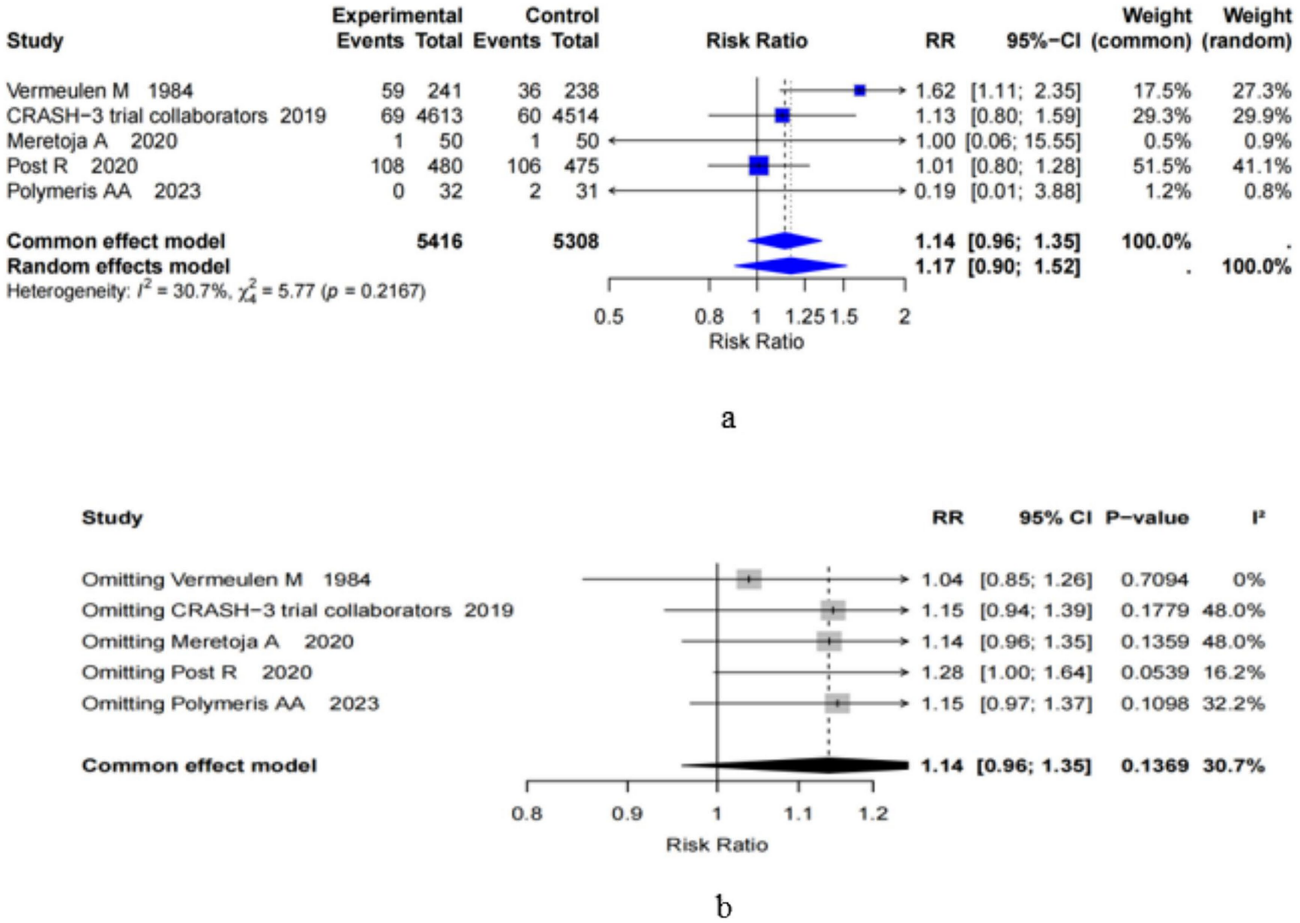
Forest plot of thromboembolic events. **(a)** Thromboembolic events. **(b)** Leave-one-out sensitivity analysis for thromboembolic events.

Five studies (10, 12, 14, 26, 27) comprising 5,416 patients in the TXA group and 5,308 in the control group reported thromboembolic events. Pooled analysis using a random-effects model showed that TXA did not significantly increase the risk of thromboembolic events (RR 1.17, 95% CI 0.90–1.52; Figure 6a). Low to moderate heterogeneity was observed across studies (*I*^2^ = 30.7%, *χ*^2^ = 5.77, *p* = 0.217), indicating generally consistent findings. Results from the fixed-effects model were directionally consistent with the primary analysis (pooled RR 1.14, 95% CI 0.96–1.35); neither model reached statistical significance, suggesting that this outcome was stable across statistical methods. Leave-one-out sensitivity analysis showed that after sequentially excluding individual studies, pooled effect estimates ranged from 1.04 to 1.28, with limited overall variation and no obvious change in the direction of effect. Notably, exclusion of the Post study (14) yielded a pooled result approaching statistical significance (RR 1.28, 95% CI 1.00–1.64; Figure 6b), suggesting that individual studies may exert some influence on the overall estimate, though this was insufficient to alter the primary conclusion. Funnel plots showed no clear asymmetry.

## 4 GRADE assessment

Table 2 presents the Summary of Findings for all primary and secondary outcomes. The certainty of evidence was rated as high for short-term mortality and functional dependence or death; moderate for long-term mortality, functional independence, and thromboembolic events; and low for rebleeding. The high certainty for the two primary outcomes of mortality and disability was supported by large sample sizes, low heterogeneity, and low risk of bias in the included trials. Rebleeding was rated as low certainty, reflecting substantial inconsistency (*I*^2^ = 72%), model dependency, and imprecision.

**Table 2:** GRADE summary of findings for key outcomes.

Outcomes	Studies (participants)	Relative effect (95% CI)	Anticipated absolute effects (per 1,000 patients)	Certainty	Comments
			Risk with placebo	Risk with TXA (95% CI)	
Short-term mortality (≤30 days)	4 RCTs (12,605)	RR 0.93 (0.86–1.00)	150	140 (129–150)	High
Long-term mortality (>30 days)	6 RCTs (4,120)	RR 1.02 (0.91–1.13)	250	255 (228–283)	Moderate
Functional independence (mRS 0–3)	6 RCTs (4,120)	RR 0.97 (0.92–1.03)	500	485 (460–515)	Moderate
Functional dependence or death (mRS 4–6)	7 RCTs (13,247)	RR 1.01 (0.96–1.07)	400	404 (384–428)	High
Rebleeding	6 RCTs (4,120)	RR 0.81 (0.59–1.11)	150	122 (89–167)	Low
Thromboembolic events	5 RCTs (10,724)	RR 1.17 (0.90–1.52)	30	35 (27–46)	Moderate

CI, confidence interval; mRS, modified Rankin Scale; RCT, randomized controlled trial; RR, relative risk; TXA, tranexamic acid. Assumed risks represent the estimated median control group risk across included studies for each outcome.

## 5 Discussion

This systematic review and meta-analysis included seven high-quality randomized controlled trials involving over 12,000 patients with hemorrhagic acute brain injury. The analysis showed that TXA did not significantly reduce short-term or long-term mortality, nor did it improve functional independence or reduce functional dependence. Regarding safety, TXA did not significantly increase the risk of thromboembolic events. For rebleeding, the pooled random-effects estimate crossed the line of no effect and exhibited substantial heterogeneity (*I*^2^ = 72%). Although the point estimate numerically favored TXA, the wide confidence interval and notable model dependency (Fixed-effect RR 0.83, 95% CI 0.74–0.93; Random-effect RR 0.81, 95% CI 0.59–1.11) preclude any definitive conclusion regarding the effect of TXA on rebleeding. Furthermore, the GRADE assessment provides important context for interpreting these findings. The certainty of evidence was judged to be high for the primary outcomes of short-term mortality and functional dependence or death, indicating that further research is unlikely to change the conclusion that TXA does not confer clinically meaningful benefits on these endpoints in an unselected population. The certainty was moderate for long-term mortality, functional independence, and thromboembolic events, reflecting minor concerns regarding risk of bias in one contributing trial or imprecision in the effect estimate. Notably, the evidence for rebleeding was rated as low certainty due to substantial heterogeneity (*I*^2^ = 72%), model dependency, and a wide confidence interval. This underscores that the observed numerical reduction in rebleeding is unreliable and should not be interpreted as a definitive treatment effect.

In the field of traumatic brain injury, the CRASH-3 trial suggested that the benefit of TXA may be confined to patients with mild-to-moderate injury (GCS 9–15), with no apparent efficacy in severe TBI (GCS 3–8) (10). The pooled data in this study showed no improvement in overall mortality, consistent with the core conclusion of CRASH-3. Early mortality in severe TBI is predominantly driven by irreversible diffuse axonal injury or brain herniation rather than by the control of intracranial hemorrhage alone. Under these circumstances, even if TXA inhibits fibrinolysis, the primary mechanical injury and cascading neurotoxic reactions that have already occurred lie beyond the biological window amenable to pharmacological salvage.

In intracerebral hemorrhage, the TICH-2 trial demonstrated that TXA reduced hematoma expansion by approximately 1.4 mL, yet no improvement was observed in 90 day modified Rankin Scale scores (11). The STOP-AUST and STOP-MSU trials, despite employing computed tomography angiography spot sign enrichment or ultra-early administration within 2 h of symptom onset, also failed to achieve positive results regarding hematoma control or neurological recovery (12, 13). A meta-analysis of individual patient data by Al-Shahi Salman et al. (3) identified hematoma expansion as a strong predictor of death and poor outcome in intracerebral hemorrhage; however, the 1-2 mL reduction in bleeding volume achieved with TXA may be insufficient to alter the secondary brain injury cascade once initiated.

In aneurysmal subarachnoid hemorrhage, the ULTRA trial showed that ultra-early short-course TXA reduced rebleeding but did not improve 6-month functional outcomes, a finding attributable to the unique pathophysiology of aneurysmal subarachnoid hemorrhage (14). Although rebleeding is an important contributor to early mortality, overall prognosis is more substantially influenced by the combined effects of delayed cerebral ischemia, hydrocephalus, and systemic complications.

The failure of the hemostatic effect of TXA in hemorrhagic acute brain injury to translate into significant improvement in patient-important outcomes can be explained at the pathophysiological level. The severity of primary injury, mass effect of the hematoma, blood–brain barrier disruption, perihematomal edema, neuro-inflammatory cascades, and delayed cerebral ischemia collectively drive the final outcome. Studies have shown that thrombin, iron ions, and hemoglobin degradation products after intracerebral hemorrhage can trigger extensive neurotoxic and inflammatory reactions (28); once these cascading events are initiated, merely reducing or controlling hematoma volume is unlikely to reverse disease progression. Furthermore, the efficacy of TXA is markedly time-dependent. An individual patient data meta-analysis of 40,138 patients with bleeding by Gayet-Ageron and colleagues confirmed that the treatment effect of TXA declines sharply with treatment delay, with survival decreasing by approximately 10% for every 15 min delay, and no discernible benefit after 3 h (7). In intracerebral hemorrhage, the STOP-MSU trial achieved a median time to administration of 105 min, yet still failed to improve hematoma expansion (13), suggesting that active bleeding may occur predominantly within minutes to 1 h after symptom onset. Consequently, even with aggressive efforts to minimize treatment delay in clinical trials, the optimal therapeutic window may already have been missed.

Our findings further highlight the limitations of surrogate endpoints in intracranial hemorrhage research. The underlying premise of surrogate endpoints such as hematoma expansion, rebleeding, or radiological hemorrhage progression—that reducing bleeding volume improves prognosis—was tacitly assumed by early investigators. However, studies ranging from recombinant activated factor VII to TXA have demonstrated that although inhibition of fibrinolysis can reduce hematoma expansion, this has not been accompanied by statistically significant improvements in neurological function or reductions in mortality (29, 30). Future trials should abandon designs using hematoma expansion as the primary endpoint and instead adopt mortality or the modified Rankin Scale as core endpoints, while exploring composite endpoints that integrate neurological deterioration and the need for surgical intervention to more accurately reflect patient-important outcomes.

Regarding safety, this study found no significant increase in the risk of thromboembolic events with TXA (RR 1.17, 95% CI 0.90–1.52), consistent with the safety profiles reported in the CRASH-3, TICH-2, and ULTRA trials (10, 11, 14). However, the upper limit of the confidence interval suggests that a clinically relevant increase in risk cannot be entirely excluded, particularly in patients receiving concomitant prothrombin complex concentrate or antiplatelet therapy. Notably, sensitivity analysis indicated that after excluding one study, the pooled estimate for cerebrovascular occlusion events approached statistical significance (RR 1.28, 95% CI 1.00–1.64), reminding us that in the specific context of aneurysmal subarachnoid hemorrhage, the prothrombotic tendency of TXA may compound the risk of delayed cerebral ischemia, warranting vigilance in clinical practice (31).

The inclusion of traumatic brain injury, intracerebral hemorrhage, and aneurysmal subarachnoid hemorrhage in a single meta-analysis represents a methodological innovation, yet it entails inherent challenges. The three conditions differ substantially in etiology, natural history, time window for intervention, and standard of care, making clinical heterogeneity unavoidable. Traumatic brain injury involves diffuse axonal injury and contusional hemorrhage, with a relatively narrow window for anti-fibrinolytic intervention; intracerebral hemorrhage is characterized by spontaneous rupture of small vessels and hematoma expansion driven by ongoing bleeding and blood–brain barrier disruption; aneurysmal subarachnoid hemorrhage results from aneurysm rupture, with subsequent risks of rebleeding, delayed cerebral ischemia, and hydrocephalus. We employed random-effects models to address anticipated heterogeneity and verified the robustness of the primary outcomes through leave-one-out sensitivity analysis. However, it must be acknowledged that the limited number of included studies, combined with variations in TXA dosing regimens across trials, may have diluted the true treatment signal in specific subgroups. Moreover, with fewer than 10 studies included, the power of funnel plot assessment for publication bias is limited, and reliable meta-regression to explore sources of heterogeneity was not feasible. The substantial heterogeneity observed for the rebleeding outcome (*I*^2^ = 72%) precisely reflects the complexity of hemorrhagic brain injury across different etiologies, suggesting that future clinical application of TXA in intracranial hemorrhage should move away from pursuing homogeneous, unified conclusions and instead shift toward pathophysiology-based precision interventions.

This study has several limitations. First, heterogeneity among studies in the definitions of neurological outcomes and follow-up time points constitutes an important limitation for the interpretation of outcomes. Most trials used the modified Rankin Scale to assess functional outcomes, whereas the earlier Vermeulen trial employed the Glasgow Outcome Scale. In addition, assessment timing varied by etiology: 28 days in the CRASH-3 trial, 90 days in the TICH-2, STOP-AUST, STOP-MSU, and TICH-NOAC trials, and 3–6 months in the ULTRA and Vermeulen trials. Second, the inclusion of three types of hemorrhagic intracranial injuries with substantially different etiologies in a single meta-analysis inevitably introduced clinical heterogeneity. Although random-effects models and leave-one-out sensitivity analysis partially mitigated this issue, subgroup analyses were not feasible due to the limited number of trials for each disease category. Third, variations in TXA dosing regimens (dose, duration, and route of administration) across trials may have diluted the true treatment effect in specific subgroups. Fourth, although the cumulative sample size exceeded 12,000 patients, the number of independent randomized comparisons was limited to seven trials. This distinction is methodologically important: a large cumulative sample size does not equate to a large number of independent comparisons, and the limited number of trials constrains the statistical power for detecting publication bias through funnel plot asymmetry and precludes reliable meta-regression to explore sources of heterogeneity.

This study carries clear implications for current clinical practice: pending more robust positive evidence, TXA should not be routinely administered to the broad population of patients with hemorrhagic acute brain injury. Existing guidelines maintain a reserved stance regarding the recommendation of TXA in acute brain injury, and neither the European Stroke Organization nor the European Association of Neurosurgical Societies has incorporated TXA into standard recommendations. By integrating evidence across disease categories, this study further reinforces this position. Nevertheless, an overall negative result does not equate to absolute ineffectiveness: the potential signals of benefit for TXA in mild-to-moderate traumatic brain injury, specific hyperfibrinolytic phenotypes, or ultra-early treatment windows remain to be validated through precision-oriented research. Future studies should focus on the following directions: first, individualized stratification based on fibrinolytic phenotypes defined by admission thromboelastography or rotational thromboelastometry, with TXA initiated only in hyperfibrinolytic subgroups; second, exploring the feasibility of prehospital administration to shift the treatment window to within 1 h of symptom onset; third, developing composite endpoints that integrate hematoma expansion, neurological deterioration, and the need for surgical intervention, replacing isolated radiological volume measurements.

## 6 Conclusion

This meta-analysis included seven high-quality randomized controlled trials comprising more than 12,000 patients with hemorrhagic acute brain injury. The results showed that TXA failed to significantly reduce all-cause mortality in either the short or long term, and did not improve neurological outcomes. Although intracranial rebleeding rates showed a numerical decrease, the confidence intervals included the null value, and substantial between-study heterogeneity precluded definitive conclusions. The incidence of thromboembolic events showed no statistically significant difference between groups, suggesting that TXA did not introduce additional safety concerns in this population. These findings indicate that while TXA may attenuate intracranial hemorrhage progression to some extent, this hemostatic effect did not translate into meaningful improvements in mortality or functional prognosis. Radiological control of hematoma expansion does not parallel ultimate functional recovery in a straightforward manner. Therefore, this updated body of high-quality evidence does not support the routine use of TXA in unselected patients with hemorrhagic acute brain injury.

## Data Availability

The original contributions presented in the study are included in the article/Supplementary material, further inquiries can be directed to the corresponding author.

## Footnotes

1
https://www.gradepro.org
