# Supplementary material for: Tranexamic acid for neurological outcomes and mortality in adults with hemorrhagic acute brain injury: an updated systematic review and meta-analysis of randomized controlled trials
Source: Front Neurol. 2026 Jul 31;17:1902987. doi: 10.3389/fneur.2026.1902987 (PMC13473429; doi:10.3389/fneur.2026.1902987)
Supplement: Supplementary file 1 [file Supplementary_file_2.DOCX]

**Supplementary File 2: Search strategy**

Source Pubmed

Search date: 2026.04.06

Result:170

| Search query | Results |
| --- | --- |
| (((((((("Brain Injuries, Traumatic"[Mesh]) OR "Craniocerebral Trauma"[Mesh]) OR (((((((((((((((((((((((((((((((((((((((((((((((((((((((((((((((((((((((((((((((((((((((((((Craniocerebral Traumas[Title/Abstract]) OR (Trauma, Craniocerebral[Title/Abstract])) OR (Traumas, Craniocerebral[Title/Abstract])) OR (Head Injuries[Title/Abstract])) OR (Injuries, Head[Title/Abstract])) OR (Head Injury[Title/Abstract])) OR (Injury, Head[Title/Abstract])) OR (Trauma, Head[Title/Abstract])) OR (Craniocerebral Injuries[Title/Abstract])) OR (Craniocerebral Injury[Title/Abstract])) OR (Injury, Craniocerebral[Title/Abstract])) OR (Injuries, Craniocerebral[Title/Abstract])) OR (Head Trauma[Title/Abstract])) OR (Head Traumas[Title/Abstract])) OR (Traumas, Head[Title/Abstract])) OR (Frontal Region Trauma[Title/Abstract])) OR (Frontal Region Traumas[Title/Abstract])) OR (Region Trauma, Frontal[Title/Abstract])) OR (Region Traumas, Frontal[Title/Abstract])) OR (Trauma, Frontal Region[Title/Abstract])) OR (Traumas, Frontal Region[Title/Abstract])) OR (Forehead Trauma[Title/Abstract])) OR (Forehead Traumas[Title/Abstract])) OR (Trauma, Forehead[Title/Abstract])) OR (Traumas, Forehead[Title/Abstract])) OR (Occipital Region Trauma[Title/Abstract])) OR (Occipital Region Traumas[Title/Abstract])) OR (Region Trauma, Occipital[Title/Abstract])) OR (Region Traumas, Occipital[Title/Abstract])) OR (Trauma, Occipital Region[Title/Abstract])) OR (Traumas, Occipital Region[Title/Abstract])) OR (Occipital Trauma[Title/Abstract])) OR (Occipital Traumas[Title/Abstract])) OR (Trauma, Occipital[Title/Abstract])) OR (Traumas, Occipital[Title/Abstract])) OR (Parietal Region Trauma[Title/Abstract])) OR (Parietal Region Traumas[Title/Abstract])) OR (Region Trauma, Parietal[Title/Abstract])) OR (Region Traumas, Parietal[Title/Abstract])) OR (Trauma, Parietal Region[Title/Abstract])) OR (Traumas, Parietal Region[Title/Abstract])) OR (Temporal Region Trauma[Title/Abstract])) OR (Temporal Region Traumas[Title/Abstract])) OR (Traumas, Temporal Region[Title/Abstract])) OR (Trauma, Temporal Region[Title/Abstract])) OR (Crushing Skull Injury[Title/Abstract])) OR (Crushing Skull Injuries[Title/Abstract])) OR (Skull Injuries, Crushing[Title/Abstract])) OR (Skull Injury, Crushing[Title/Abstract])) OR (Head Injuries, Multiple[Title/Abstract])) OR (Head Injury, Multiple[Title/Abstract])) OR (Injuries, Multiple Head[Title/Abstract])) OR (Injury, Multiple Head[Title/Abstract])) OR (Multiple Head Injury[Title/Abstract])) OR (Multiple Head Injuries[Title/Abstract])) OR (Head Injury, Minor[Title/Abstract])) OR (Head Injuries, Minor[Title/Abstract])) OR (Injuries, Minor Head[Title/Abstract])) OR (Injury, Minor Head[Title/Abstract])) OR (Minor Head Injuries[Title/Abstract])) OR (Minor Head Injury[Title/Abstract])) OR (Head Injury, Open[Title/Abstract])) OR (Head Injuries, Open[Title/Abstract])) OR (Injuries, Open Head[Title/Abstract])) OR (Injury, Open Head[Title/Abstract])) OR (Open Head Injuries[Title/Abstract])) OR (Open Head Injury[Title/Abstract])) OR (Head Injury, Superficial[Title/Abstract])) OR (Head Injuries, Superficial[Title/Abstract])) OR (Injuries, Superficial Head[Title/Abstract])) OR (Injury, Superficial Head[Title/Abstract])) OR (Superficial Head Injuries[Title/Abstract])) OR (Superficial Head Injury[Title/Abstract])) OR (Brain Injury, Traumatic[Title/Abstract])) OR (Traumatic Brain Injuries[Title/Abstract])) OR (Trauma, Brain[Title/Abstract])) OR (Brain Trauma[Title/Abstract])) OR (Brain Traumas[Title/Abstract])) OR (Traumas, Brain[Title/Abstract])) OR (Traumatic Brain Injury[Title/Abstract])) OR (Encephalopathy, Traumatic[Title/Abstract])) OR (Encephalopathies, Traumatic[Title/Abstract])) OR (Traumatic Encephalopathies[Title/Abstract])) OR (Injury, Brain, Traumatic[Title/Abstract])) OR (Traumatic Encephalopathy[Title/Abstract])) OR (TBIs (Traumatic Brain Injuries[Title/Abstract]))) OR (TBI (Traumatic Brain Injuries[Title/Abstract]))) OR (TBI (Traumatic Brain Injury[Title/Abstract]))) OR (TBI[Title/Abstract])) OR (brain lesion[Title/Abstract])) OR (brain damage[Title/Abstract]))) OR ("Subarachnoid Hemorrhage"[Mesh])) OR (((((((((((((((((((((((((((((((Hemorrhage, Subarachnoid[Title/Abstract]) OR (Hemorrhages, Subarachnoid[Title/Abstract])) OR (Subarachnoid Hemorrhages[Title/Abstract])) OR (SAH (Subarachnoid Hemorrhage[Title/Abstract]))) OR (SAHs (Subarachnoid Hemorrhage[Title/Abstract]))) OR (Perinatal Subarachnoid Hemorrhage[Title/Abstract])) OR (Hemorrhage, Perinatal Subarachnoid[Title/Abstract])) OR (Hemorrhages, Perinatal Subarachnoid[Title/Abstract])) OR (Perinatal Subarachnoid Hemorrhages[Title/Abstract])) OR (Subarachnoid Hemorrhage, Perinatal[Title/Abstract])) OR (Subarachnoid Hemorrhages, Perinatal[Title/Abstract])) OR (Subarachnoid Hemorrhage, Aneurysmal[Title/Abstract])) OR (Aneurysmal Subarachnoid Hemorrhage[Title/Abstract])) OR (Aneurysmal Subarachnoid Hemorrhages[Title/Abstract])) OR (Hemorrhage, Aneurysmal Subarachnoid[Title/Abstract])) OR (Hemorrhages, Aneurysmal Subarachnoid[Title/Abstract])) OR (Subarachnoid Hemorrhages, Aneurysmal[Title/Abstract])) OR (Subarachnoid Hemorrhage, Spontaneous[Title/Abstract])) OR (Hemorrhage, Spontaneous Subarachnoid[Title/Abstract])) OR (Hemorrhages, Spontaneous Subarachnoid[Title/Abstract])) OR (Spontaneous Subarachnoid Hemorrhage[Title/Abstract])) OR (Spontaneous Subarachnoid Hemorrhages[Title/Abstract])) OR (Subarachnoid Hemorrhages, Spontaneous[Title/Abstract])) OR (Subarachnoid Hemorrhage, Intracranial[Title/Abstract])) OR (Hemorrhage, Intracranial Subarachnoid[Title/Abstract])) OR (Hemorrhages, Intracranial Subarachnoid[Title/Abstract])) OR (Intracranial Subarachnoid Hemorrhage[Title/Abstract])) OR (Intracranial Subarachnoid Hemorrhages[Title/Abstract])) OR (Subarachnoid Hemorrhages, Intracranial[Title/Abstract])) OR (subarachnoid haemorrhage[Title/Abstract])) OR (subarachnoid bleeding[Title/Abstract]))) OR ("Cerebral Hemorrhage"[Mesh])) OR ((((((((((((((((((((((((Hemorrhage, Cerebral[Title/Abstract]) OR (Cerebral Hemorrhages[Title/Abstract])) OR (Hemorrhages, Cerebral[Title/Abstract])) OR (Intracerebral Hemorrhage[Title/Abstract])) OR (Hemorrhage, Intracerebral[Title/Abstract])) OR (Hemorrhages, Intracerebral[Title/Abstract])) OR (Intracerebral Hemorrhages[Title/Abstract])) OR (Hemorrhage, Cerebrum[Title/Abstract])) OR (Cerebrum Hemorrhage[Title/Abstract])) OR (Cerebrum Hemorrhages[Title/Abstract])) OR (Hemorrhages, Cerebrum[Title/Abstract])) OR (Brain Hemorrhage, Cerebral[Title/Abstract])) OR (Brain Hemorrhages, Cerebral[Title/Abstract])) OR (Cerebral Brain Hemorrhage[Title/Abstract])) OR (Cerebral Brain Hemorrhages[Title/Abstract])) OR (Hemorrhage, Cerebral Brain[Title/Abstract])) OR (Hemorrhages, Cerebral Brain[Title/Abstract])) OR (Cerebral Parenchymal Hemorrhage[Title/Abstract])) OR (Cerebral Parenchymal Hemorrhages[Title/Abstract])) OR (Hemorrhage, Cerebral Parenchymal[Title/Abstract])) OR (Hemorrhages, Cerebral Parenchymal[Title/Abstract])) OR (Parenchymal Hemorrhage, Cerebral[Title/Abstract])) OR (Parenchymal Hemorrhages, Cerebral[Title/Abstract])) OR (intraparenchymal[Title/Abstract]))) AND (("Tranexamic Acid"[Mesh]) OR ((((((((((((AMCA[Title/Abstract]) OR (AMCHA[Title/Abstract])) OR (t-AMCHA[Title/Abstract])) OR (trans-4-(Aminomethyl)cyclohexanecarboxylic Acid[Title/Abstract])) OR (Cyklokapron[Title/Abstract])) OR (Ugurol[Title/Abstract])) OR (Transamin[Title/Abstract])) OR (KABI 2161[Title/Abstract])) OR (Amchafibrin[Title/Abstract])) OR (Anvitoff[Title/Abstract])) OR (Spotof[Title/Abstract])) OR (Exacyl[Title/Abstract])))) AND ("randomized controlled trial"[Publication Type] OR "randomized"[Title/Abstract] OR "placebo"[Title/Abstract]) | 170 |

Source: Scopus

Searched date: 2026.04.06

Result: 444

| Search query | Results |
| --- | --- |
| ( ( TITLE-ABS-KEY ( "Brain Injuries, Traumatic" ) OR TITLE-ABS-KEY ( "Craniocerebral Trauma" ) OR TITLE-ABS-KEY ( "Subarachnoid Hemorrhage" ) OR TITLE-ABS-KEY ( "Cerebral Hemorrhage" ) OR TITLE-ABS-KEY ( "Craniocerebral Traumas" ) OR TITLE-ABS-KEY ( "Trauma, Craniocerebral" ) OR TITLE-ABS-KEY ( "Traumas, Craniocerebral" ) OR TITLE-ABS-KEY ( "Head Injuries" ) OR TITLE-ABS-KEY ( "Injuries, Head" ) OR TITLE-ABS-KEY ( "Head Injury" ) OR TITLE-ABS-KEY ( "Injury, Head" ) OR TITLE-ABS-KEY ( "Trauma, Head" ) OR TITLE-ABS-KEY ( "Craniocerebral Injuries" ) OR TITLE-ABS-KEY ( "Craniocerebral Injury" ) OR TITLE-ABS-KEY ( "Injury, Craniocerebral" ) OR TITLE-ABS-KEY ( "Injuries, Craniocerebral" ) OR TITLE-ABS-KEY ( "Head Trauma" ) OR TITLE-ABS-KEY ( "Head Traumas" ) OR TITLE-ABS-KEY ( "Traumas, Head" ) OR TITLE-ABS-KEY ( "Frontal Region Trauma" ) OR TITLE-ABS-KEY ( "Frontal Region Traumas" ) OR TITLE-ABS-KEY ( "Region Trauma, Frontal" ) OR TITLE-ABS-KEY ( "Region Traumas, Frontal" ) OR TITLE-ABS-KEY ( "Trauma, Frontal Region" ) OR TITLE-ABS-KEY ( "Traumas, Frontal Region" ) OR TITLE-ABS-KEY ( "Forehead Trauma" ) OR TITLE-ABS-KEY ( "Forehead Traumas" ) OR TITLE-ABS-KEY ( "Trauma, Forehead" ) OR TITLE-ABS-KEY ( "Traumas, Forehead" ) OR TITLE-ABS-KEY ( "Occipital Region Trauma" ) OR TITLE-ABS-KEY ( "Occipital Region Traumas" ) OR TITLE-ABS-KEY ( "Region Trauma, Occipital" ) OR TITLE-ABS-KEY ( "Region Traumas, Occipital" ) OR TITLE-ABS-KEY ( "Trauma, Occipital Region" ) OR TITLE-ABS-KEY ( "Traumas, Occipital Region" ) OR TITLE-ABS-KEY ( "Occipital Trauma" ) OR TITLE-ABS-KEY ( "Occipital Traumas" ) OR TITLE-ABS-KEY ( "Trauma, Occipital" ) OR TITLE-ABS-KEY ( "Traumas, Occipital" ) OR TITLE-ABS-KEY ( "Parietal Region Trauma" ) OR TITLE-ABS-KEY ( "Parietal Region Traumas" ) OR TITLE-ABS-KEY ( "Region Trauma, Parietal" ) OR TITLE-ABS-KEY ( "Region Traumas, Parietal" ) OR TITLE-ABS-KEY ( "Trauma, Parietal Region" ) OR TITLE-ABS-KEY ( "Traumas, Parietal Region" ) OR TITLE-ABS-KEY ( "Temporal Region Trauma" ) OR TITLE-ABS-KEY ( "Temporal Region Traumas" ) OR TITLE-ABS-KEY ( "Traumas, Temporal Region" ) OR TITLE-ABS-KEY ( "Trauma, Temporal Region" ) OR TITLE-ABS-KEY ( "Crushing Skull Injury" ) OR TITLE-ABS-KEY ( "Crushing Skull Injuries" ) OR TITLE-ABS-KEY ( "Skull Injuries, Crushing" ) OR TITLE-ABS-KEY ( "Skull Injury, Crushing" ) OR TITLE-ABS-KEY ( "Head Injuries, Multiple" ) OR TITLE-ABS-KEY ( "Head Injury, Multiple" ) OR TITLE-ABS-KEY ( "Injuries, Multiple Head" ) OR TITLE-ABS-KEY ( "Injury, Multiple Head" ) OR TITLE-ABS-KEY ( "Multiple Head Injury" ) OR TITLE-ABS-KEY ( "Multiple Head Injuries" ) OR TITLE-ABS-KEY ( "Head Injury, Minor" ) OR TITLE-ABS-KEY ( "Head Injuries, Minor" ) OR TITLE-ABS-KEY ( "Injuries, Minor Head" ) OR TITLE-ABS-KEY ( "Injury, Minor Head" ) OR TITLE-ABS-KEY ( "Minor Head Injuries" ) OR TITLE-ABS-KEY ( "Minor Head Injury" ) OR TITLE-ABS-KEY ( "Head Injury, Open" ) OR TITLE-ABS-KEY ( "Head Injuries, Open" ) OR TITLE-ABS-KEY ( "Injuries, Open Head" ) OR TITLE-ABS-KEY ( "Injury, Open Head" ) OR TITLE-ABS-KEY ( "Open Head Injuries" ) OR TITLE-ABS-KEY ( "Open Head Injury" ) OR TITLE-ABS-KEY ( "Head Injury, Superficial" ) OR TITLE-ABS-KEY ( "Head Injuries, Superficial" ) OR TITLE-ABS-KEY ( "Injuries, Superficial Head" ) OR TITLE-ABS-KEY ( "Injury, Superficial Head" ) OR TITLE-ABS-KEY ( "Superficial Head Injuries" ) OR TITLE-ABS-KEY ( "Superficial Head Injury" ) OR TITLE-ABS-KEY ( "Brain Injury, Traumatic" ) OR TITLE-ABS-KEY ( "Traumatic Brain Injuries" ) OR TITLE-ABS-KEY ( "Trauma, Brain" ) OR TITLE-ABS-KEY ( "Brain Trauma" ) OR TITLE-ABS-KEY ( "Brain Traumas" ) OR TITLE-ABS-KEY ( "Traumas, Brain" ) OR TITLE-ABS-KEY ( "Traumatic Brain Injury" ) OR TITLE-ABS-KEY ( "Encephalopathy, Traumatic" ) OR TITLE-ABS-KEY ( "Encephalopathies, Traumatic" ) OR TITLE-ABS-KEY ( "Traumatic Encephalopathies" ) OR TITLE-ABS-KEY ( "Injury, Brain, Traumatic" ) OR TITLE-ABS-KEY ( "Traumatic Encephalopathy" ) OR TITLE-ABS-KEY ( "TBIs (Traumatic Brain Injuries)" ) OR TITLE-ABS-KEY ( "TBI (Traumatic Brain Injuries)" ) OR TITLE-ABS-KEY ( "TBI (Traumatic Brain Injury)" ) OR TITLE-ABS-KEY ( "TBI" ) OR TITLE-ABS-KEY ( "brain lesion" ) OR TITLE-ABS-KEY ( "brain damage" ) OR TITLE-ABS-KEY ( "Hemorrhage, Subarachnoid" ) OR TITLE-ABS-KEY ( "Hemorrhages, Subarachnoid" ) OR TITLE-ABS-KEY ( "Subarachnoid Hemorrhages" ) OR TITLE-ABS-KEY ( "SAH (Subarachnoid Hemorrhage)" ) OR TITLE-ABS-KEY ( "SAHs (Subarachnoid Hemorrhage)" ) OR TITLE-ABS-KEY ( "Perinatal Subarachnoid Hemorrhage" ) OR TITLE-ABS-KEY ( "Hemorrhage, Perinatal Subarachnoid" ) OR TITLE-ABS-KEY ( "Hemorrhages, Perinatal Subarachnoid" ) OR TITLE-ABS-KEY ( "Perinatal Subarachnoid Hemorrhages" ) OR TITLE-ABS-KEY ( "Subarachnoid Hemorrhage, Perinatal" ) OR TITLE-ABS-KEY ( "Subarachnoid Hemorrhages, Perinatal" ) OR TITLE-ABS-KEY ( "Subarachnoid Hemorrhage, Aneurysmal" ) OR TITLE-ABS-KEY ( "Aneurysmal Subarachnoid Hemorrhage" ) OR TITLE-ABS-KEY ( "Aneurysmal Subarachnoid Hemorrhages" ) OR TITLE-ABS-KEY ( "Hemorrhage, Aneurysmal Subarachnoid" ) OR TITLE-ABS-KEY ( "Hemorrhages, Aneurysmal Subarachnoid" ) OR TITLE-ABS-KEY ( "Subarachnoid Hemorrhages, Aneurysmal" ) OR TITLE-ABS-KEY ( "Subarachnoid Hemorrhage, Spontaneous" ) OR TITLE-ABS-KEY ( "Hemorrhage, Spontaneous Subarachnoid" ) OR TITLE-ABS-KEY ( "Hemorrhages, Spontaneous Subarachnoid" ) OR TITLE-ABS-KEY ( "Spontaneous Subarachnoid Hemorrhage" ) OR TITLE-ABS-KEY ( "Spontaneous Subarachnoid Hemorrhages" ) OR TITLE-ABS-KEY ( "Subarachnoid Hemorrhages, Spontaneous" ) OR TITLE-ABS-KEY ( "Subarachnoid Hemorrhage, Intracranial" ) OR TITLE-ABS-KEY ( "Hemorrhage, Intracranial Subarachnoid" ) OR TITLE-ABS-KEY ( "Hemorrhages, Intracranial Subarachnoid" ) OR TITLE-ABS-KEY ( "Intracranial Subarachnoid Hemorrhage" ) OR TITLE-ABS-KEY ( "Intracranial Subarachnoid Hemorrhages" ) OR TITLE-ABS-KEY ( "Subarachnoid Hemorrhages, Intracranial" ) OR TITLE-ABS-KEY ( "subarachnoid haemorrhage" ) OR TITLE-ABS-KEY ( "subarachnoid bleeding" ) OR TITLE-ABS-KEY ( "Hemorrhage, Cerebral" ) OR TITLE-ABS-KEY ( "Cerebral Hemorrhages" ) OR TITLE-ABS-KEY ( "Hemorrhages, Cerebral" ) OR TITLE-ABS-KEY ( "Intracerebral Hemorrhage" ) OR TITLE-ABS-KEY ( "Hemorrhage, Intracerebral" ) OR TITLE-ABS-KEY ( "Hemorrhages, Intracerebral" ) OR TITLE-ABS-KEY ( "Intracerebral Hemorrhages" ) OR TITLE-ABS-KEY ( "Hemorrhage, Cerebrum" ) OR TITLE-ABS-KEY ( "Cerebrum Hemorrhage" ) OR TITLE-ABS-KEY ( "Cerebrum Hemorrhages" ) OR TITLE-ABS-KEY ( "Hemorrhages, Cerebrum" ) OR TITLE-ABS-KEY ( "Brain Hemorrhage, Cerebral" ) OR TITLE-ABS-KEY ( "Brain Hemorrhages, Cerebral" ) OR TITLE-ABS-KEY ( "Cerebral Brain Hemorrhage" ) OR TITLE-ABS-KEY ( "Cerebral Brain Hemorrhages" ) OR TITLE-ABS-KEY ( "Hemorrhage, Cerebral Brain" ) OR TITLE-ABS-KEY ( "Hemorrhages, Cerebral Brain" ) OR TITLE-ABS-KEY ( "Cerebral Parenchymal Hemorrhage" ) OR TITLE-ABS-KEY ( "Cerebral Parenchymal Hemorrhages" ) OR TITLE-ABS-KEY ( "Hemorrhage, Cerebral Parenchymal" ) OR TITLE-ABS-KEY ( "Hemorrhages, Cerebral Parenchymal" ) OR TITLE-ABS-KEY ( "Parenchymal Hemorrhage, Cerebral" ) OR TITLE-ABS-KEY ( "Parenchymal Hemorrhages, Cerebral" ) OR TITLE-ABS-KEY ( "intraparenchymal" ) ) ) AND ( ( TITLE-ABS-KEY ( "Tranexamic Acid" ) OR TITLE-ABS-KEY ( "AMCA" ) OR TITLE-ABS-KEY ( "AMCHA" ) OR TITLE-ABS-KEY ( "t-AMCHA" ) OR TITLE-ABS-KEY ( "trans-4-(Aminomethyl)cyclohexanecarboxylic Acid" ) OR TITLE-ABS-KEY ( "Cyklokapron" ) OR TITLE-ABS-KEY ( "Ugurol" ) OR TITLE-ABS-KEY ( "Transamin" ) OR TITLE-ABS-KEY ( "KABI 2161" ) OR TITLE-ABS-KEY ( "Amchafibrin" ) OR TITLE-ABS-KEY ( "Anvitoff" ) OR TITLE-ABS-KEY ( "Spotof" ) OR TITLE-ABS-KEY ( "Exacyl" ) ) ) AND ( ( TITLE-ABS-KEY ( " randomized AND controlled AND trial" ) OR TITLE-ABS-KEY ( "randomized" ) OR TITLE-ABS-KEY ( "placebo" ) ) ) | 444 |

Source: Web of science

Searched date: 2026.04.06

Result: 230

| Step | Search query | Results |
| --- | --- | --- |
| #4 | #1 AND #2 AND #3 | 230 |
| #3 | randomized controlled trial (Topic) OR randomized (Topic) OR placebo (Topic) | 870,227 |
| #2 | Tranexamic Acid (Topic) OR AMCA (Topic) OR AMCHA (Topic) OR t-AMCHA (Topic) OR trans-4-(Aminomethyl)cyclohexanecarboxylic Acid (Topic) OR Cyklokapron (Topic) OR Ugurol (Topic) OR Transamin (Topic) OR KABI 2161 (Topic) OR Amchafibrin (Topic) OR Anvitoff (Topic) OR Spotof (Topic) OR Exacyl (Topic) | 8,439 |
| #1 | Brain Injuries, Traumatic (Topic) OR Craniocerebral Trauma (Topic) OR Subarachnoid Hemorrhage (Topic) OR Cerebral Hemorrhage (Topic) OR Craniocerebral Traumas (Topic) OR Trauma, Craniocerebral (Topic) OR Traumas, Craniocerebral (Topic) OR Head Injuries (Topic) OR Injuries, Head (Topic) OR Head Injury (Topic) OR Injury, Head (Topic) OR Trauma, Head (Topic) OR Craniocerebral Injuries (Topic) OR Craniocerebral Injury (Topic) OR Injury, Craniocerebral (Topic) OR Injuries, Craniocerebral (Topic) OR Head Trauma (Topic) OR Head Traumas (Topic) OR Traumas, Head (Topic) OR Frontal Region Trauma (Topic) OR Frontal Region Traumas (Topic) OR Region Trauma, Frontal (Topic) OR Region Traumas, Frontal (Topic) OR Trauma, Frontal Region (Topic) OR Traumas, Frontal Region (Topic) OR Forehead Trauma (Topic) OR Forehead Traumas (Topic) OR Trauma, Forehead (Topic) OR Traumas, Forehead (Topic) OR Occipital Region Trauma (Topic) OR Occipital Region Traumas (Topic) OR Region Trauma, Occipital (Topic) OR Region Traumas, Occipital (Topic) OR Trauma, Occipital Region (Topic) OR Traumas, Occipital Region (Topic) OR Occipital Trauma (Topic) OR Occipital Traumas (Topic) OR Trauma, Occipital (Topic) OR Traumas, Occipital (Topic) OR Parietal Region Trauma (Topic) OR Parietal Region Traumas (Topic) OR Region Trauma, Parietal (Topic) OR Region Traumas, Parietal (Topic) OR Trauma, Parietal Region (Topic) OR Traumas, Parietal Region (Topic) OR Temporal Region Trauma (Topic) OR Temporal Region Traumas (Topic) OR Traumas, Temporal Region (Topic) OR Trauma, Temporal Region (Topic) OR Crushing Skull Injury (Topic) OR Crushing Skull Injuries (Topic) OR Skull Injuries, Crushing (Topic) OR Skull Injury, Crushing (Topic) OR Head Injuries, Multiple (Topic) OR Head Injury, Multiple (Topic) OR Injuries, Multiple Head (Topic) OR Injury, Multiple Head (Topic) OR Multiple Head Injury (Topic) OR Multiple Head Injuries (Topic) OR Head Injury, Minor (Topic) OR Head Injuries, Minor (Topic) OR Injuries, Minor Head (Topic) OR Injury, Minor Head (Topic) OR Minor Head Injuries (Topic) OR Minor Head Injury (Topic) OR Head Injury, Open (Topic) OR Head Injuries, Open (Topic) OR Injuries, Open Head (Topic) OR Injury, Open Head (Topic) OR Open Head Injuries (Topic) OR Open Head Injury (Topic) OR Head Injury, Superficial (Topic) OR Head Injuries, Superficial (Topic) OR Injuries, Superficial Head (Topic) OR Injury, Superficial Head (Topic) OR Superficial Head Injuries (Topic) OR Superficial Head Injury (Topic) OR Brain Injury, Traumatic (Topic) OR Traumatic Brain Injuries (Topic) OR Trauma, Brain (Topic) OR Brain Trauma (Topic) OR Brain Traumas (Topic) OR Traumas, Brain (Topic) OR Traumatic Brain Injury (Topic) OR Encephalopathy, Traumatic (Topic) OR Encephalopathies, Traumatic (Topic) OR Traumatic Encephalopathies (Topic) OR Injury, Brain, Traumatic (Topic) OR Traumatic Encephalopathy (Topic) OR TBIs (Traumatic Brain Injuries) (Topic) OR TBI (Traumatic Brain Injuries) (Topic) OR TBI (Traumatic Brain Injury) (Topic) OR TBI (Topic) OR brain lesion (Topic) OR brain damage (Topic) OR Hemorrhage, Subarachnoid (Topic) OR Hemorrhages, Subarachnoid (Topic) OR Subarachnoid Hemorrhages (Topic) OR SAH (Subarachnoid Hemorrhage) (Topic) OR SAHs (Subarachnoid Hemorrhage) (Topic) OR Perinatal Subarachnoid Hemorrhage (Topic) OR Hemorrhage, Perinatal Subarachnoid (Topic) OR Hemorrhages, Perinatal Subarachnoid (Topic) OR Perinatal Subarachnoid Hemorrhages (Topic) OR Subarachnoid Hemorrhage, Perinatal (Topic) OR Subarachnoid Hemorrhages, Perinatal (Topic) OR Subarachnoid Hemorrhage, Aneurysmal (Topic) OR Aneurysmal Subarachnoid Hemorrhage (Topic) OR Aneurysmal Subarachnoid Hemorrhages (Topic) OR Hemorrhage, Aneurysmal Subarachnoid (Topic) OR Hemorrhages, Aneurysmal Subarachnoid (Topic) OR Subarachnoid Hemorrhages, Aneurysmal (Topic) OR Subarachnoid Hemorrhage, Spontaneous (Topic) OR Hemorrhage, Spontaneous Subarachnoid (Topic) OR Hemorrhages, Spontaneous Subarachnoid (Topic) OR Spontaneous Subarachnoid Hemorrhage (Topic) OR Spontaneous Subarachnoid Hemorrhages (Topic) OR Subarachnoid Hemorrhages, Spontaneous (Topic) OR Subarachnoid Hemorrhage, Intracranial (Topic) OR Hemorrhage, Intracranial Subarachnoid (Topic) OR Hemorrhages, Intracranial Subarachnoid (Topic) OR Intracranial Subarachnoid Hemorrhage (Topic) OR Intracranial Subarachnoid Hemorrhages (Topic) OR Subarachnoid Hemorrhages, Intracranial (Topic) OR subarachnoid haemorrhage (Topic) OR subarachnoid bleeding (Topic) OR Hemorrhage, Cerebral (Topic) OR Cerebral Hemorrhages (Topic) OR Hemorrhages, Cerebral (Topic) OR Intracerebral Hemorrhage (Topic) OR Hemorrhage, Intracerebral (Topic) OR Hemorrhages, Intracerebral (Topic) OR Intracerebral Hemorrhages (Topic) OR Hemorrhage, Cerebrum (Topic) OR Cerebrum Hemorrhage (Topic) OR Cerebrum Hemorrhages (Topic) OR Hemorrhages, Cerebrum (Topic) OR Brain Hemorrhage, Cerebral (Topic) OR Brain Hemorrhages, Cerebral (Topic) OR Cerebral Brain Hemorrhage (Topic) OR Cerebral Brain Hemorrhages (Topic) OR Hemorrhage, Cerebral Brain (Topic) OR Hemorrhages, Cerebral Brain (Topic) OR Cerebral Parenchymal Hemorrhage (Topic) OR Cerebral Parenchymal Hemorrhages (Topic) OR Hemorrhage, Cerebral Parenchymal (Topic) OR Hemorrhages, Cerebral Parenchymal (Topic) OR Parenchymal Hemorrhage, Cerebral (Topic) OR Parenchymal Hemorrhages, Cerebral (Topic) OR intraparenchymal (Topic) | 269,016 |

Source: Embase

Searched date: 2026.04.08

Result: 282

| Step | Search query | Results |
| --- | --- | --- |
| #8 | #3 AND #6 AND #7 | 282 |
| #7 | 'random':ab,ti,kw OR 'placebo':ab,ti,kw OR 'double blind':ab,ti,kw | 1,129,789 |
| #6 | #4 OR #5 | 25,813 |
| #5 | 'amca':ab,ti,kw OR 'amcha':ab,ti,kw OR 't-amcha':ab,ti,kw OR 'trans-4-(aminomethyl)cyclohexanecarboxylic acid':ab,ti,kw OR 'cyklokapron':ab,ti,kw OR 'ugurol':ab,ti,kw OR 'transamin':ab,ti,kw OR 'kabi 2161':ab,ti,kw OR 'amchafibrin':ab,ti,kw OR 'anvitoff':ab,ti,kw OR 'spotof':ab,ti,kw OR 'exacyl':ab,ti,kw | 441，911 |
| #4 | 'tranexamic acid'/exp | 25,315 |
| #3 | #1OR #2 | 420,023 |
| #2 | 'craniocerebral traumas':ab,ti,kw OR 'trauma,craniocerebral':ab,ti,kw OR 'traumas, craniocerebral':ab,ti,kw OR 'head injuries':ab,ti,kw OR 'injuries, head':ab,ti,kw OR 'head injury':ab,ti,kw OR 'injury, head':ab,ti,kw OR 'trauma, head':ab,ti,kw OR 'craniocerebral injuries':ab,ti,kw OR 'craniocerebral injury':ab,ti,kw OR 'injury, craniocerebral':ab,ti,kw OR 'injuries, craniocerebral':ab,ti,kw OR 'head trauma':ab,ti,kw OR 'head traumas':ab,ti,kw OR 'traumas, head':ab,ti,kw OR 'frontal region trauma':ab,ti,kw OR 'frontal region traumas':ab,ti,kw OR 'region trauma, frontal':ab,ti,kw OR 'region traumas, frontal':ab,ti,kw OR 'trauma, frontal region':ab,ti,kw OR 'traumas, frontal region':ab,ti,kw OR 'forehead trauma':ab,ti,kw OR 'forehead traumas':ab,ti,kw OR 'trauma, forehead':ab,ti,kw OR 'traumas, forehead':ab,ti,kw OR 'occipital region trauma':ab,ti,kw OR 'occipital region traumas':ab,ti,kw OR 'region trauma, occipital':ab,ti,kw OR 'region traumas, occipital':ab,ti,kw OR 'trauma, occipital region':ab,ti,kw OR 'traumas, occipital region':ab,ti,kw OR 'occipital trauma':ab,ti,kw OR 'occipital traumas':ab,ti,kw OR 'trauma, occipital':ab,ti,kw OR 'traumas, occipital':ab,ti,kw OR 'parietal region trauma':ab,ti,kw OR 'parietal region traumas':ab,ti,kw OR 'region trauma, parietal':ab,ti,kw OR 'region traumas, parietal':ab,ti,kw OR 'trauma, parietal region':ab,ti,kw OR 'traumas, parietal region':ab,ti,kw OR 'temporal region trauma':ab,ti,kw OR 'temporal region traumas':ab,ti,kw OR 'traumas, temporal region':ab,ti,kw OR 'trauma, temporal region':ab,ti,kw OR 'crushing skull injury':ab,ti,kw OR 'crushing skull injuries':ab,ti,kw OR 'skull injuries,  crushing':ab,ti,kw OR 'skull injury, crushing':ab,ti,kw OR 'head injuries, multiple':ab,ti,kw OR 'head injury, multiple':ab,ti,kw OR 'injuries, multiple head':ab,ti,kw OR 'injury, multiple head':ab,ti,kw OR 'multiple head injury':ab,ti,kw OR 'multiple head injuries':ab,ti,kw OR 'head injury, minor':ab,ti,kw OR 'head injuries, minor':ab,ti,kw OR 'injuries, minor head':ab,ti,kw OR 'injury, minor head':ab,ti,kw OR 'minor head injuries':ab,ti,kw OR 'minor head injury':ab,ti,kw OR 'head injury, open':ab,ti,kw OR 'head injuries, open':ab,ti,kw OR 'injuries, open head':ab,ti,kw OR 'injury, open head':ab,ti,kw OR 'open head injuries':ab,ti,kw OR 'open head injury':ab,ti,kw OR 'head injury, superficial':ab,ti,kw OR 'head injuries, superficial':ab,ti,kw OR 'injuries, superficial head':ab,ti,kw OR 'injury, superficial head':ab,ti,kw OR 'superficial head injuries':ab,ti,kw OR 'superficial head injury':ab,ti,kw OR 'brain injury, traumatic':ab,ti,kw OR 'traumatic brain injuries':ab,ti,kw OR 'trauma, brain':ab,ti,kw OR 'brain trauma':ab,ti,kw OR 'brain traumas':ab,ti,kw OR 'traumas, brain':ab,ti,kw OR 'traumatic brain injury':ab,ti,kw OR 'encephalopathy, traumatic':ab,ti,kw OR 'encephalopathies, traumatic':ab,ti,kw OR 'traumatic encephalopathies':ab,ti,kw OR 'injury, brain, traumatic':ab,ti,kw OR 'traumatic encephalopathy':ab,ti,kw OR 'tbis (traumatic brain injuries)':ab,ti,kw OR 'tbi (traumatic brain injuries)':ab,ti,kw OR 'tbi (traumatic brain injury)':ab,ti,kw OR 'tbi':ab,ti,kw OR 'brain lesion':ab,ti,kw OR 'brain damage':ab,ti,kw OR 'hemorrhage, subarachnoid':ab,ti,kw OR 'hemorrhages, subarachnoid':ab,ti,kw OR 'subarachnoid hemorrhages':ab,ti,kw OR 'sah (subarachnoid hemorrhage)':ab,ti,kw OR 'sahs (subarachnoid hemorrhage)':ab,ti,kw OR 'perinatal subarachnoid hemorrhage':ab,ti,kw OR 'hemorrhage, perinatal subarachnoid':ab,ti,kw OR 'hemorrhages, perinatal subarachnoid':ab,ti,kw OR 'perinatal subarachnoid hemorrhages':ab,ti,kw OR 'subarachnoid hemorrhage, perinatal':ab,ti,kw OR 'subarachnoid hemorrhages, perinatal':ab,ti,kw OR 'subarachnoid hemorrhage, aneurysmal':ab,ti,kw OR 'aneurysmal subarachnoid hemorrhages':ab,ti,kw OR 'aneurysmal subarachnoid hemorrhage':ab,ti,kw OR 'hemorrhage, aneurysmal subarachnoid':ab,ti,kw OR 'hemorrhages, aneurysmal subarachnoid':ab,ti,kw OR 'subarachnoid hemorrhages, aneurysmal':ab,ti,kw OR 'subarachnoid hemorrhage, spontaneous':ab,ti,kw OR 'hemorrhages, spontaneous subarachnoid':ab,ti,kw OR 'hemorrhage, spontaneous subarachnoid':ab,ti,kw OR 'spontaneous subarachnoid hemorrhage':ab,ti,kw OR 'spontaneous subarachnoid hemorrhages':ab,ti,kw OR 'subarachnoid hemorrhages, spontaneous':ab,ti,kw OR 'subarachnoid hemorrhage, intracranial':ab,ti,kw OR 'hemorrhage, intracranial subarachnoid':ab,ti,kw OR 'hemorrhages, intracranial subarachnoid':ab,ti,kw OR 'intracranial subarachnoid hemorrhage':ab,ti,kw OR 'intracranial subarachnoid hemorrhages':ab,ti,kw OR 'subarachnoid hemorrhages,intracranial':ab,ti,kw OR 'subarachnoid haemorrhage':ab,ti,kw OR 'subarachnoid bleeding':ab,ti,kw OR 'hemorrhage,cerebral':ab,ti,kw OR 'cerebral hemorrhages':ab,ti,kw OR 'hemorrhages, cerebral':ab,ti,kw OR 'intracerebral hemorrhage':ab,ti,kw OR 'hemorrhage, intracerebral':ab,ti,kw OR 'hemorrhages,intracerebral':ab,ti,kw OR 'intracerebral hemorrhages':ab,ti,kw OR 'hemorrhage, cerebrum':ab,ti,kw OR 'cerebrum hemorrhage':ab,ti,kw OR 'cerebrum hemorrhages':ab,ti,kw OR 'hemorrhages, cerebrum':ab,ti,kw OR 'brain hemorrhage, cerebral':ab,ti,kw OR 'brain hemorrhages, cerebral':ab,ti,kw OR 'cerebral brain hemorrhage':ab,ti,kw OR 'cerebral brain hemorrhages':ab,ti,kw OR 'hemorrhage, cerebral brain':ab,ti,kw OR 'hemorrhages, cerebral brain':ab,ti,kw OR 'cerebral parenchymal hemorrhage':ab,ti,kw OR 'cerebral parenchymal hemorrhages':ab,ti,kw OR 'hemorrhage, cerebral parenchymal':ab,ti,kw OR 'hemorrhages, cerebral parenchymal':ab,ti,kw OR 'parenchymal hemorrhage, cerebral':ab,ti,kw OR 'parenchymal hemorrhages, cerebral':ab,ti,kw OR 'intraparenchymal':ab,ti,kw | 244,427 |
| #1 | 'traumatic brain injury'/exp OR 'subarachnoid hemorrhage'/exp OR 'brain hemorrhage'/exp | 299,909 |

Source: Cochrane Central Register of Controlled Trials (CENTRAL)

Searched date: 2026.03.26

Result:170 (Including 3 reviews)

| Step | Search query | Results |
| --- | --- | --- |
| #9 | #5 AND #8 | 170 |
| #8 | #6 OR #7 | 2,153 |
| #7 | (AMCA):ti,ab,kw OR (AMCHA):ti,ab,kw OR (t-AMCHA):ti,ab,kw OR (Cyklokapron):ti,ab,kw OR (Ugurol):ti,ab,kw OR (Transamin):ti,ab,kw OR (KABI 2161):ti,ab,kw OR (Amchafibrin):ti,ab,kw OR (Anvitoff):ti,ab,kw OR (Spotof):ti,ab,kw OR (Exacyl):ti,ab,kw | 133 |
| #6 | MeSH descriptor: [Tranexamic Acid] explode all trees | 2,053 |
| #5 | #1 OR #2 OR #3 OR #4 | 27,971 |
| #4 | (Craniocerebral Traumas):ti,ab,kw OR (Trauma, Craniocerebral):ti,ab,kw OR (Traumas, Craniocerebral):ti,ab,kw OR (Head Injuries):ti,ab,kw OR (Injuries, Head):ti,ab,kw OR (Head Injury):ti,ab,kw OR (Injury, Head):ti,ab,kw OR (Trauma, Head):ti,ab,kw OR (Craniocerebral Injuries):ti,ab,kw OR (Craniocerebral Injury):ti,ab,kw OR (Injury, Craniocerebral):ti,ab,kw OR (Injuries, Craniocerebral):ti,ab,kw OR (Head Trauma):ti,ab,kw OR (Head Traumas):ti,ab,kw OR (Traumas, Head):ti,ab,kw OR (Frontal Region Trauma):ti,ab,kw OR (Frontal Region Traumas):ti,ab,kw OR (Region Trauma, Frontal):ti,ab,kw OR (Region Traumas, Frontal):ti,ab,kw OR (Trauma, Frontal Region):ti,ab,kw OR (Traumas, Frontal Region):ti,ab,kw OR (Forehead Trauma):ti,ab,kw OR (Forehead Traumas):ti,ab,kw OR (Trauma, Forehead):ti,ab,kw OR (Traumas, Forehead):ti,ab,kw OR (Occipital Region Trauma):ti,ab,kw OR (Occipital Region Traumas):ti,ab,kw OR (Region Trauma, Occipital):ti,ab,kw OR (Region Traumas, Occipital):ti,ab,kw OR (Trauma, Occipital Region):ti,ab,kw OR (Traumas, Occipital Region):ti,ab,kw OR (Occipital Trauma):ti,ab,kw OR (Occipital Traumas):ti,ab,kw OR (Trauma, Occipital):ti,ab,kw OR (Traumas, Occipital):ti,ab,kw OR (Parietal Region Trauma):ti,ab,kw OR (Parietal Region Traumas):ti,ab,kw OR (Region Trauma, Parietal):ti,ab,kw OR (Region Traumas, Parietal):ti,ab,kw OR (Trauma, Parietal Region):ti,ab,kw OR (Traumas, Parietal Region):ti,ab,kw OR (Temporal Region Trauma):ti,ab,kw OR (Temporal Region Traumas):ti,ab,kw OR (Traumas, Temporal Region):ti,ab,kw OR (Trauma, Temporal Region):ti,ab,kw OR (Crushing Skull Injury):ti,ab,kw OR (Crushing Skull Injuries):ti,ab,kw OR (Skull Injuries, Crushing):ti,ab,kw OR (Skull Injury, Crushing):ti,ab,kw OR (Head Injuries, Multiple):ti,ab,kw OR (Head Injury, Multiple):ti,ab,kw OR (Injuries, Multiple Head):ti,ab,kw OR (Injury, Multiple Head):ti,ab,kw OR (Multiple Head Injury):ti,ab,kw OR (Multiple Head Injuries):ti,ab,kw OR (Head Injury, Minor):ti,ab,kw OR (Head Injuries, Minor):ti,ab,kw OR (Injuries, Minor Head):ti,ab,kw OR (Injury, Minor Head):ti,ab,kw OR (Minor Head Injuries):ti,ab,kw OR (Minor Head Injury):ti,ab,kw OR (Head Injury, Open):ti,ab,kw OR (Head Injuries, Open):ti,ab,kw OR (Injuries, Open Head):ti,ab,kw OR (Injury, Open Head):ti,ab,kw OR (Open Head Injuries):ti,ab,kw OR (Open Head Injury):ti,ab,kw OR (Head Injury, Superficial):ti,ab,kw OR (Head Injuries, Superficial):ti,ab,kw OR (Injury, Superficial Head):ti,ab,kw OR (Injuries, Superficial Head):ti,ab,kw OR (Superficial Head Injuries):ti,ab,kw OR (Superficial Head Injury):ti,ab,kw OR (Brain Injury, Traumatic):ti,ab,kw OR (Traumatic Brain Injuries):ti,ab,kw OR (Trauma, Brain):ti,ab,kw OR (Brain Trauma):ti,ab,kw OR (Brain Traumas):ti,ab,kw OR (Traumas, Brain):ti,ab,kw OR (Traumatic Brain Injury):ti,ab,kw OR (Encephalopathy, Traumatic):ti,ab,kw OR (Encephalopathies, Traumatic):ti,ab,kw OR (Traumatic Encephalopathies):ti,ab,kw OR (Injury, Brain, Traumatic):ti,ab,kw OR (Traumatic Encephalopathy):ti,ab,kw OR (TBIs (Traumatic Brain Injuries)):ti,ab,kw OR (TBI (Traumatic Brain Injuries)):ti,ab,kw OR (TBI (Traumatic Brain Injury)):ti,ab,kw OR (TBI):ti,ab,kw OR (brain lesion):ti,ab,kw OR (brain damage):ti,ab,kw OR (Hemorrhage, Subarachnoid):ti,ab,kw OR (Hemorrhages, Subarachnoid):ti,ab,kw OR (Subarachnoid Hemorrhages):ti,ab,kw OR (SAH (Subarachnoid Hemorrhage)):ti,ab,kw OR (SAHs (Subarachnoid Hemorrhage)):ti,ab,kw OR (Perinatal Subarachnoid Hemorrhage):ti,ab,kw OR (Hemorrhage, Perinatal Subarachnoid):ti,ab,kw OR (Hemorrhages, Perinatal Subarachnoid):ti,ab,kw OR (Perinatal Subarachnoid Hemorrhages):ti,ab,kw OR (Subarachnoid Hemorrhages, Perinatal):ti,ab,kw OR (Subarachnoid Hemorrhage, Perinatal):ti,ab,kw OR (Subarachnoid Hemorrhage, Aneurysmal):ti,ab,kw OR (Aneurysmal Subarachnoid Hemorrhage):ti,ab,kw OR (Aneurysmal Subarachnoid Hemorrhages):ti,ab,kw OR (Hemorrhage, Aneurysmal Subarachnoid):ti,ab,kw OR (Hemorrhages, Aneurysmal Subarachnoid):ti,ab,kw OR (Subarachnoid Hemorrhages, Aneurysmal):ti,ab,kw OR (Subarachnoid Hemorrhage, Spontaneous):ti,ab,kw OR (Hemorrhage, Spontaneous Subarachnoid):ti,ab,kw OR (Hemorrhages, Spontaneous Subarachnoid):ti,ab,kw OR (Spontaneous Subarachnoid Hemorrhage):ti,ab,kw OR (Spontaneous Subarachnoid Hemorrhages):ti,ab,kw OR (Subarachnoid Hemorrhages, Spontaneous):ti,ab,kw OR (Subarachnoid Hemorrhage, Intracranial):ti,ab,kw OR (Hemorrhage, Intracranial Subarachnoid):ti,ab,kw OR (Hemorrhages, Intracranial Subarachnoid):ti,ab,kw OR (Intracranial Subarachnoid Hemorrhage):ti,ab,kw OR (Intracranial Subarachnoid Hemorrhages):ti,ab,kw OR (Subarachnoid Hemorrhages, Intracranial):ti,ab,kw OR (subarachnoid haemorrhage):ti,ab,kw OR (subarachnoid bleeding):ti,ab,kw OR (Hemorrhage, Cerebral):ti,ab,kw OR (Cerebral Hemorrhages):ti,ab,kw OR (Hemorrhages, Cerebral):ti,ab,kw OR (Intracerebral Hemorrhage):ti,ab,kw OR (Hemorrhage, Intracerebral):ti,ab,kw OR (Hemorrhages, Intracerebral):ti,ab,kw OR (Intracerebral Hemorrhages):ti,ab,kw OR (Hemorrhage, Cerebrum):ti,ab,kw OR (Cerebrum Hemorrhage):ti,ab,kw OR (Cerebrum Hemorrhages):ti,ab,kw OR (Hemorrhages, Cerebrum):ti,ab,kw OR (Brain Hemorrhage, Cerebral):ti,ab,kw OR (Brain Hemorrhages, Cerebral):ti,ab,kw OR (Cerebral Brain Hemorrhage):ti,ab,kw OR (Cerebral Brain Hemorrhages):ti,ab,kw OR (Hemorrhage, Cerebral Brain):ti,ab,kw OR (Hemorrhages, Cerebral Brain):ti,ab,kw OR (Cerebral Parenchymal Hemorrhage):ti,ab,kw OR (Cerebral Parenchymal Hemorrhages):ti,ab,kw OR (Hemorrhage, Cerebral Parenchymal):ti,ab,kw OR (Hemorrhages, Cerebral Parenchymal):ti,ab,kw OR (Parenchymal Hemorrhage, Cerebral):ti,ab,kw OR (Parenchymal Hemorrhages, Cerebral):ti,ab,kw OR (intraparenchymal):ti,ab,kw | 27,747 |
| #3 | MeSH descriptor: [Cerebral Hemorrhage] explode all trees | 1,689 |
| #2 | MeSH descriptor: [Subarachnoid Hemorrhage] explode all trees | 893 |
| #1 | MeSH descriptor: [Brain Injuries, Traumatic] explode all trees | 1,985 |
