# Supplementary material for: Tranexamic acid for neurological outcomes and mortality in adults with hemorrhagic acute brain injury: an updated systematic review and meta-analysis of randomized controlled trials
Source: Front Neurol. 2026 Jul 31;17:1902987. doi: 10.3389/fneur.2026.1902987 (PMC13473429; doi:10.3389/fneur.2026.1902987)
Supplement: Supplementary file 2 [file Supplementary_file_3.DOCX]

**Supplementary file 3. ROB2**

**Figure S1. Risk of bias assessment 2.0 for included studies**

As percentage (intention-to-treat)

|  | Low risk | Some concerns | High risk |
| --- | --- | --- | --- |
| Randomization process | 100 | 0 | 0 |
| Deviations from intended interventions | 85.7 | 14.3 | 0 |
| Mising outcome data | 100 | 0 | 0 |
| Measurement of the outcome | 100 | 0 | 0 |
| Selection of the reported result | 100 | 0 | 0 |
| Overall Bias | 85.7 | 14.3 | 0 |

**Table S1. Risk of bias assessment signaling question 2.0 for included studies**

| **Experimental** | tranexamic acid | **Comparator** | placebo | |
| --- | --- | --- | --- | --- |
| **Assessor 1** | Zhengyi Zhang | **Assessor 2** | Zilin He | |
| **Outcome** | Mortality, neurological functional recovery,cerebrovascular occlusion events,rebleeding rate | | | |
| **Domain** | **Signalling question for the study of Vermeulen M 1984** | | | **Response** |
| **Bias arising from the randomization process** | 1.1 Was the allocation sequence random? | | | Y |
|  | 1.2 Was the allocation sequence concealed until participants were enrolled and assigned to interventions? | | | Y |
|  | 1.3 Did baseline differences between intervention groups suggest a problem with the randomization process? | | | PN |
|  | **Risk of bias judgement** | | | **Low** |
| **Bias due to deviations from intended interventions** | 2.1.Were participants aware of their assigned intervention during the trial? | | | N |
|  | 2.2.Were carers and people delivering the interventions aware of participants' assigned intervention during the trial? | | | PN |
|  | 2.3. If Y/PY/NI to 2.1 or 2.2: Were there deviations from the intended intervention that arose because of the experimental context? | | | NA |
|  | 2.4 If Y/PY to 2.3: Were these deviations likely to have affected the outcome? | | | NA |
|  | 2.5. If Y/PY/NI to 2.4: Were these deviations from intended intervention balanced between groups? | | | NA |
|  | 2.6 Was an appropriate analysis used to estimate the effect of assignment to intervention? | | | PY |
|  | 2.7 If N/PN/NI to 2.6: Was there potential for a substantial impact (on the result) of the failure to analyse participants in the group to which they were randomized? | | | NA |
|  | **Risk of bias judgement** | | | **Low** |
| **Bias due to missing outcome data** | 3.1 Were data for this outcome available for all, or nearly all, participants randomized? | | | Y |
|  | 3.2 If N/PN/NI to 3.1: Is there evidence that result was not biased by missing outcome data? | | | NA |
|  | 3.3 If N/PN to 3.2: Could missingness in the outcome depend on its true value? | | | NA |
|  | 3.4 If Y/PY/NI to 3.3: Is it likely that missingness in the outcome depended on its true value? | | | NA |
|  | **Risk of bias judgement** | | | **Low** |
| **Bias in measurement of the outcome** | 4.1 Was the method of measuring the outcome inappropriate? | | | N |
|  | 4.2 Could measurement or ascertainment of the outcome have differed between intervention groups? | | | N |
|  | 4.3 Were outcome assessors aware of the intervention received by study participants? | | | N |
|  | 4.4 If Y/PY/NI to 4.3: Could assessment of the outcome have been influenced by knowledge of intervention received? | | | NA |
|  | 4.5 If Y/PY/NI to 4.4: Is it likely that assessment of the outcome was influenced by knowledge of intervention received? | | | NA |
|  | **Risk of bias judgement** | | | **Low** |
| **Bias in selection of the reported result** | 5.1 Were the data that produced this result analysed in accordance with a pre-specified analysis plan that was finalized before unblinded outcome data were available for analysis? | | | PY |
|  | 5.2 ... multiple eligible outcome measurements (e.g. scales, definitions, time points) within the outcome domain? | | | N |
|  | 5.3 ... multiple eligible analyses of the data? | | | N |
|  | **Risk of bias judgement** | | | **LOW** |
| **Overall bias** | **Risk of bias judgement** | | | **LOW** |
| **Domain** | **Signalling question for the study of Sprigg N 2018** | | | **Response** |
| **Bias arising from the randomization process** | 1.1 Was the allocation sequence random? | | | Y |
|  | 1.2 Was the allocation sequence concealed until participants were enrolled and assigned to interventions? | | | Y |
|  | 1.3 Did baseline differences between intervention groups suggest a problem with the randomization process? | | | N |
|  | **Risk of bias judgement** | | | **Low** |
| **Bias due to deviations from intended interventions** | 2.1.Were participants aware of their assigned intervention during the trial? | | | N |
|  | 2.2.Were carers and people delivering the interventions aware of participants' assigned intervention during the trial? | | | N |
|  | 2.3. If Y/PY/NI to 2.1 or 2.2: Were there deviations from the intended intervention that arose because of the experimental context? | | | NA |
|  | 2.4 If Y/PY to 2.3: Were these deviations likely to have affected the outcome? | | | NA |
|  | 2.5. If Y/PY/NI to 2.4: Were these deviations from intended intervention balanced between groups? | | | NA |
|  | 2.6 Was an appropriate analysis used to estimate the effect of assignment to intervention? | | | Y |
|  | 2.7 If N/PN/NI to 2.6: Was there potential for a substantial impact (on the result) of the failure to analyse participants in the group to which they were randomized? | | | NA |
|  | **Risk of bias judgement** | | | **Low** |
| **Bias due to missing outcome data** | 3.1 Were data for this outcome available for all, or nearly all, participants randomized? | | | Y |
|  | 3.2 If N/PN/NI to 3.1: Is there evidence that result was not biased by missing outcome data? | | | NA |
|  | 3.3 If N/PN to 3.2: Could missingness in the outcome depend on its true value? | | | NA |
|  | 3.4 If Y/PY/NI to 3.3: Is it likely that missingness in the outcome depended on its true value? | | | NA |
|  | **Risk of bias judgement** | | | **Low** |
| **Bias in measurement of the outcome** | 4.1 Was the method of measuring the outcome inappropriate? | | | N |
|  | 4.2 Could measurement or ascertainment of the outcome have differed between intervention groups? | | | N |
|  | 4.3 Were outcome assessors aware of the intervention received by study participants? | | | N |
|  | 4.4 If Y/PY/NI to 4.3: Could assessment of the outcome have been influenced by knowledge of intervention received? | | | NA |
|  | 4.5 If Y/PY/NI to 4.4: Is it likely that assessment of the outcome was influenced by knowledge of intervention received? | | | NA |
|  | **Risk of bias judgement** | | | **Low** |
| **Bias in selection of the reported result** | 5.1 Were the data that produced this result analysed in accordance with a pre-specified analysis plan that was finalized before unblinded outcome data were available for analysis? | | | Y |
|  | 5.2 ... multiple eligible outcome measurements (e.g. scales, definitions, time points) within the outcome domain? | | | N |
|  | 5.3 ... multiple eligible analyses of the data? | | | N |
|  | **Risk of bias judgement** | | | **Low** |
| **Overall bias** | **Risk of bias judgement** | | | **Low** |
| **Domain** | **Signalling question for the study of CRASH-3 trial collaborators 2019** | | | **Response** |
| **Bias arising from the randomization process** | 1.1 Was the allocation sequence random? | | | Y |
|  | 1.2 Was the allocation sequence concealed until participants were enrolled and assigned to interventions? | | | Y |
|  | 1.3 Did baseline differences between intervention groups suggest a problem with the randomization process? | | | N |
|  | **Risk of bias judgement** | | | **Low** |
| **Bias due to deviations from intended interventions** | 2.1.Were participants aware of their assigned intervention during the trial? | | | N |
|  | 2.2.Were carers and people delivering the interventions aware of participants' assigned intervention during the trial? | | | Y |
|  | 2.3. If Y/PY/NI to 2.1 or 2.2: Were there deviations from the intended intervention that arose because of the experimental context? | | | N |
|  | 2.4 If Y/PY to 2.3: Were these deviations likely to have affected the outcome? | | | NA |
|  | 2.5. If Y/PY/NI to 2.4: Were these deviations from intended intervention balanced between groups? | | | NA |
|  | 2.6 Was an appropriate analysis used to estimate the effect of assignment to intervention? | | | Y |
|  | 2.7 If N/PN/NI to 2.6: Was there potential for a substantial impact (on the result) of the failure to analyse participants in the group to which they were randomized? | | | NA |
|  | **Risk of bias judgement** | | | **Low** |
| **Bias due to missing outcome data** | 3.1 Were data for this outcome available for all, or nearly all, participants randomized? | | | Y |
|  | 3.2 If N/PN/NI to 3.1: Is there evidence that result was not biased by missing outcome data? | | | NA |
|  | 3.3 If N/PN to 3.2: Could missingness in the outcome depend on its true value? | | | NA |
|  | 3.4 If Y/PY/NI to 3.3: Is it likely that missingness in the outcome depended on its true value? | | | NA |
|  | **Risk of bias judgement** | | | **Low** |
| **Bias in measurement of the outcome** | 4.1 Was the method of measuring the outcome inappropriate? | | | N |
|  | 4.2 Could measurement or ascertainment of the outcome have differed between intervention groups? | | | N |
|  | 4.3 Were outcome assessors aware of the intervention received by study participants? | | | N |
|  | 4.4 If Y/PY/NI to 4.3: Could assessment of the outcome have been influenced by knowledge of intervention received? | | | NA |
|  | 4.5 If Y/PY/NI to 4.4: Is it likely that assessment of the outcome was influenced by knowledge of intervention received? | | | NA |
|  | **Risk of bias judgement** | | | **Low** |
| **Bias in selection of the reported result** | 5.1 Were the data that produced this result analysed in accordance with a pre-specified analysis plan that was finalized before unblinded outcome data were available for analysis? | | | Y |
|  | 5.2 ... multiple eligible outcome measurements (e.g. scales, definitions, time points) within the outcome domain? | | | N |
|  | 5.3 ... multiple eligible analyses of the data? | | | N |
|  | **Risk of bias judgement** | | | **Low** |
| **Overall bias** | **Risk of bias judgement** | | | **Low** |
| **Domain** | **Signalling question for the study of Meretoja A 2020** | | | **Response** |
| **Bias arising from the randomization process** | 1.1 Was the allocation sequence random? | | | Y |
|  | 1.2 Was the allocation sequence concealed until participants were enrolled and assigned to interventions? | | | Y |
|  | 1.3 Did baseline differences between intervention groups suggest a problem with the randomization process? | | | PN |
|  | **Risk of bias judgement** | | | **Low** |
| **Bias due to deviations from intended interventions** | 2.1.Were participants aware of their assigned intervention during the trial? | | | N |
|  | 2.2.Were carers and people delivering the interventions aware of participants' assigned intervention during the trial? | | | N |
|  | 2.3. If Y/PY/NI to 2.1 or 2.2: Were there deviations from the intended intervention that arose because of the experimental context? | | | NA |
|  | 2.4 If Y/PY to 2.3: Were these deviations likely to have affected the outcome? | | | NA |
|  | 2.5. If Y/PY/NI to 2.4: Were these deviations from intended intervention balanced between groups? | | | NA |
|  | 2.6 Was an appropriate analysis used to estimate the effect of assignment to intervention? | | | PY |
|  | 2.7 If N/PN/NI to 2.6: Was there potential for a substantial impact (on the result) of the failure to analyse participants in the group to which they were randomized? | | | NA |
|  | **Risk of bias judgement** | | | **Low** |
| **Bias due to missing outcome data** | 3.1 Were data for this outcome available for all, or nearly all, participants randomized? | | | Y |
|  | 3.2 If N/PN/NI to 3.1: Is there evidence that result was not biased by missing outcome data? | | | NA |
|  | 3.3 If N/PN to 3.2: Could missingness in the outcome depend on its true value? | | | NA |
|  | 3.4 If Y/PY/NI to 3.3: Is it likely that missingness in the outcome depended on its true value? | | | NA |
|  | **Risk of bias judgement** | | | **Low** |
| **Bias in measurement of the outcome** | 4.1 Was the method of measuring the outcome inappropriate? | | | N |
|  | 4.2 Could measurement or ascertainment of the outcome have differed between intervention groups? | | | N |
|  | 4.3 Were outcome assessors aware of the intervention received by study participants? | | | N |
|  | 4.4 If Y/PY/NI to 4.3: Could assessment of the outcome have been influenced by knowledge of intervention received? | | | NA |
|  | 4.5 If Y/PY/NI to 4.4: Is it likely that assessment of the outcome was influenced by knowledge of intervention received? | | | NA |
|  | **Risk of bias judgement** | | | **Low** |
| **Bias in selection of the reported result** | 5.1 Were the data that produced this result analysed in accordance with a pre-specified analysis plan that was finalized before unblinded outcome data were available for analysis? | | | Y |
|  | 5.2 ... multiple eligible outcome measurements (e.g. scales, definitions, time points) within the outcome domain? | | | N |
|  | 5.3 ... multiple eligible analyses of the data? | | | N |
|  | **Risk of bias judgement** | | | **Low** |
| **Overall bias** | **Risk of bias judgement** | | | **Low** |
| **Domain** | **Signalling question for the study of Post R 2020** | | | **Response** |
| **Bias arising from the randomization process** | 1.1 Was the allocation sequence random? | | | Y |
|  | 1.2 Was the allocation sequence concealed until participants were enrolled and assigned to interventions? | | | Y |
|  | 1.3 Did baseline differences between intervention groups suggest a problem with the randomization process? | | | N |
|  | **Risk of bias judgement** | | | **Low** |
| **Bias due to deviations from intended interventions** | 2.1.Were participants aware of their assigned intervention during the trial? | | | Y |
|  | 2.2.Were carers and people delivering the interventions aware of participants' assigned intervention during the trial? | | | PY |
|  | 2.3. If Y/PY/NI to 2.1 or 2.2: Were there deviations from the intended intervention that arose because of the experimental context? | | | PY |
|  | 2.4 If Y/PY to 2.3: Were these deviations likely to have affected the outcome? | | | N |
|  | 2.5. If Y/PY/NI to 2.4: Were these deviations from intended intervention balanced between groups? | | | NA |
|  | 2.6 Was an appropriate analysis used to estimate the effect of assignment to intervention? | | | Y |
|  | 2.7 If N/PN/NI to 2.6: Was there potential for a substantial impact (on the result) of the failure to analyse participants in the group to which they were randomized? | | | NA |
|  | **Risk of bias judgement** | | | **Some Concerns** |
| **Bias due to missing outcome data** | 3.1 Were data for this outcome available for all, or nearly all, participants randomized? | | | Y |
|  | 3.2 If N/PN/NI to 3.1: Is there evidence that result was not biased by missing outcome data? | | | NA |
|  | 3.3 If N/PN to 3.2: Could missingness in the outcome depend on its true value? | | | NA |
|  | 3.4 If Y/PY/NI to 3.3: Is it likely that missingness in the outcome depended on its true value? | | | NA |
|  | **Risk of bias judgement** | | | **Low** |
| **Bias in measurement of the outcome** | 4.1 Was the method of measuring the outcome inappropriate? | | | N |
|  | 4.2 Could measurement or ascertainment of the outcome have differed between intervention groups? | | | N |
|  | 4.3 Were outcome assessors aware of the intervention received by study participants? | | | N |
|  | 4.4 If Y/PY/NI to 4.3: Could assessment of the outcome have been influenced by knowledge of intervention received? | | | NA |
|  | 4.5 If Y/PY/NI to 4.4: Is it likely that assessment of the outcome was influenced by knowledge of intervention received? | | | NA |
|  | **Risk of bias judgement** | | | **Low** |
| **Bias in selection of the reported result** | 5.1 Were the data that produced this result analysed in accordance with a pre-specified analysis plan that was finalized before unblinded outcome data were available for analysis? | | | Y |
|  | 5.2 ... multiple eligible outcome measurements (e.g. scales, definitions, time points) within the outcome domain? | | | N |
|  | 5.3 ... multiple eligible analyses of the data? | | | N |
|  | **Risk of bias judgement** | | | **Some Concerns** |
| **Overall bias** | **Risk of bias judgement** | | | **Some Concerns** |
| **Domain** | **Signalling question for the study of Polymeris AA 2023** | | | **Response** |
| **Bias arising from the randomization process** | 1.1 Was the allocation sequence random? | | | Y |
|  | 1.2 Was the allocation sequence concealed until participants were enrolled and assigned to interventions? | | | Y |
|  | 1.3 Did baseline differences between intervention groups suggest a problem with the randomization process? | | | PN |
|  | **Risk of bias judgement** | | | **Low** |
| **Bias due to deviations from intended interventions** | 2.1.Were participants aware of their assigned intervention during the trial? | | | N |
|  | 2.2.Were carers and people delivering the interventions aware of participants' assigned intervention during the trial? | | | N |
|  | 2.3. If Y/PY/NI to 2.1 or 2.2: Were there deviations from the intended intervention that arose because of the experimental context? | | | NA |
|  | 2.4 If Y/PY to 2.3: Were these deviations likely to have affected the outcome? | | | NA |
|  | 2.5. If Y/PY/NI to 2.4: Were these deviations from intended intervention balanced between groups? | | | NA |
|  | 2.6 Was an appropriate analysis used to estimate the effect of assignment to intervention? | | | Y |
|  | 2.7 If N/PN/NI to 2.6: Was there potential for a substantial impact (on the result) of the failure to analyse participants in the group to which they were randomized? | | | NA |
|  | **Risk of bias judgement** | | | **Low** |
| **Bias due to missing outcome data** | 3.1 Were data for this outcome available for all, or nearly all, participants randomized? | | | Y |
|  | 3.2 If N/PN/NI to 3.1: Is there evidence that result was not biased by missing outcome data? | | | NA |
|  | 3.3 If N/PN to 3.2: Could missingness in the outcome depend on its true value? | | | NA |
|  | 3.4 If Y/PY/NI to 3.3: Is it likely that missingness in the outcome depended on its true value? | | | NA |
|  | **Risk of bias judgement** | | | **Low** |
| **Bias in measurement of the outcome** | 4.1 Was the method of measuring the outcome inappropriate? | | | N |
|  | 4.2 Could measurement or ascertainment of the outcome have differed between intervention groups? | | | N |
|  | 4.3 Were outcome assessors aware of the intervention received by study participants? | | | N |
|  | 4.4 If Y/PY/NI to 4.3: Could assessment of the outcome have been influenced by knowledge of intervention received? | | | NA |
|  | 4.5 If Y/PY/NI to 4.4: Is it likely that assessment of the outcome was influenced by knowledge of intervention received? | | | NA |
|  | **Risk of bias judgement** | | | **Low** |
| **Bias in selection of the reported result** | 5.1 Were the data that produced this result analysed in accordance with a pre-specified analysis plan that was finalized before unblinded outcome data were available for analysis? | | | Y |
|  | 5.2 ... multiple eligible outcome measurements (e.g. scales, definitions, time points) within the outcome domain? | | | N |
|  | 5.3 ... multiple eligible analyses of the data? | | | N |
|  | **Risk of bias judgement** | | | **Low** |
| **Overall bias** | **Risk of bias judgement** | | | **Low** |
| **Domain** | **Signalling question for the study of Yassi N 2024** | | | **Response** |
| **Bias arising from the randomization process** | 1.1 Was the allocation sequence random? | | | Y |
|  | 1.2 Was the allocation sequence concealed until participants were enrolled and assigned to interventions? | | | Y |
|  | 1.3 Did baseline differences between intervention groups suggest a problem with the randomization process? | | | N |
|  | **Risk of bias judgement** | | | **Low** |
| **Bias due to deviations from intended interventions** | 2.1.Were participants aware of their assigned intervention during the trial? | | | N |
|  | 2.2.Were carers and people delivering the interventions aware of participants' assigned intervention during the trial? | | | N |
|  | 2.3. If Y/PY/NI to 2.1 or 2.2: Were there deviations from the intended intervention that arose because of the experimental context? | | | NA |
|  | 2.4 If Y/PY to 2.3: Were these deviations likely to have affected the outcome? | | | NA |
|  | 2.5. If Y/PY/NI to 2.4: Were these deviations from intended intervention balanced between groups? | | | NA |
|  | 2.6 Was an appropriate analysis used to estimate the effect of assignment to intervention? | | | Y |
|  | 2.7 If N/PN/NI to 2.6: Was there potential for a substantial impact (on the result) of the failure to analyse participants in the group to which they were randomized? | | | NA |
|  | **Risk of bias judgement** | | | **Low** |
| **Bias due to missing outcome data** | 3.1 Were data for this outcome available for all, or nearly all, participants randomized? | | | Y |
|  | 3.2 If N/PN/NI to 3.1: Is there evidence that result was not biased by missing outcome data? | | | NA |
|  | 3.3 If N/PN to 3.2: Could missingness in the outcome depend on its true value? | | | NA |
|  | 3.4 If Y/PY/NI to 3.3: Is it likely that missingness in the outcome depended on its true value? | | | NA |
|  | **Risk of bias judgement** | | | **Low** |
| **Bias in measurement of the outcome** | 4.1 Was the method of measuring the outcome inappropriate? | | | N |
|  | 4.2 Could measurement or ascertainment of the outcome have differed between intervention groups? | | | N |
|  | 4.3 Were outcome assessors aware of the intervention received by study participants? | | | N |
|  | 4.4 If Y/PY/NI to 4.3: Could assessment of the outcome have been influenced by knowledge of intervention received? | | | NA |
|  | 4.5 If Y/PY/NI to 4.4: Is it likely that assessment of the outcome was influenced by knowledge of intervention received? | | | NA |
|  | **Risk of bias judgement** | | | **Low** |
| **Bias in selection of the reported result** | 5.1 Were the data that produced this result analysed in accordance with a pre-specified analysis plan that was finalized before unblinded outcome data were available for analysis? | | | Y |
|  | 5.2 ... multiple eligible outcome measurements (e.g. scales, definitions, time points) within the outcome domain? | | | N |
|  | 5.3 ... multiple eligible analyses of the data? | | | N |
|  | **Risk of bias judgement** | | | **Low** |
| **Overall bias** | **Risk of bias judgement** | | | **Low** |
