# Supplementary material for: Tranexamic acid for neurological outcomes and mortality in adults with hemorrhagic acute brain injury: an updated systematic review and meta-analysis of randomized controlled trials
Source: Front Neurol. 2026 Jul 31;17:1902987. doi: 10.3389/fneur.2026.1902987 (PMC13473429; doi:10.3389/fneur.2026.1902987)
Supplement: Supplementary file 3 [file Supplementary_file_4.DOCX]

**Supplementary File 4: Forest Plot and Sensitivity Analysis**

**Figure S1: Funnel Plot for Short-term Mortality**

**Figure S2: Funnel Plot for** long-term mortality

**Figure S3: Funnel Plot for f**unctional independence

**Figure S4: Funnel Plot for** functional dependence

**Figure S5: Funnel Plot for rebleeding**

**Figure S6: Funnel Plot for** thromboembolic events

**Figure S7:Leave-one-out Sensitivity Analysis for Short-term Mortality**

**Figure S8:Leave-one-out Sensitivity Analysis for** long-term mortality

**Figure S9:Leave-one-out Sensitivity Analysis for f**unctional independence

**Figure S10:Leave-one-out Sensitivity Analysis for** functional dependence
